# MAT2A as Key Regulator and Therapeutic Target in *MLL*r Leukemogenesis

**DOI:** 10.3390/cancers12051342

**Published:** 2020-05-24

**Authors:** Kathy-Ann Secker, Bianca Bloechl, Hildegard Keppeler, Silke Duerr-Stoerzer, Hannes Schmid, Dominik Schneidawind, Johan Jeong, Thomas Hentrich, Julia M. Schulze-Hentrich, Corina Schneidawind

**Affiliations:** 1 Department of Hematology, Oncology, Clinical Immunology and Rheumatology, University Hospital Tuebingen, 72076 Tuebingen, Germany; kathy-ann.secker@med.uni-tuebingen.de (K.-A.S.); bianca.bloechl@center1.de (B.B.); hildegard.keppeler@med.uni-tuebingen.de (H.K.); silke.duerr-stoerzer@med.uni-tuebingen.de (S.D.-S.); hannes.schmid@gmx.de (H.S.); dominik.schneidawind@med.uni-tuebingen.de (D.S.); 2 Synthego Corporation, Menlo Park, CA 94025, USA; jeong401@gmail.com; 3 Institute of Medical Genetics and Applied Genomics, University of Tuebingen, 72076 Tuebingen, Germany; thomas.hentrich@med.uni-tuebingen.de (T.H.); julia.schulze-hentrich@med.uni-tuebingen.de (J.M.S.-H.)

**Keywords:** *MLL*-rearranged leukemia, MAT2A, CRISPR/Cas9, pharmacological study

## Abstract

Epigenetic dysregulation plays a pivotal role in mixed-lineage leukemia (*MLL)* pathogenesis, therefore serving as a suitable therapeutic target. S-adenosylmethionine (SAM) is the universal methyl donor in human cells and is synthesized by methionine adenosyltransferase 2A (MAT2A), which is deregulated in different cancer types. Here, we used our human CRISPR/Cas9-*MLL*-rearranged (CRISPR/Cas9-*MLL*r) leukemia model, faithfully mimicking *MLL*r patients’ pathology with indefinite growth potential *in vitro*, to evaluate the unknown role of MAT2A. Comparable to publicly available patient data, we detected MAT2A to be significantly overexpressed in our CRISPR/Cas9-*MLL*r model compared to healthy controls. By using non-*MLL*r and *MLL*r cell lines and our model, we detected an *MLL*r-specific enhanced response to PF-9366, a new MAT2A inhibitor, and small interfering (si) RNA-mediated knockdown of *MAT2A*, by alteration of the proliferation, viability, differentiation, apoptosis, cell cycling, and histone methylation. Moreover, the combinational treatment of PF-9366 with chemotherapy or targeted therapies against the SAM-dependent methyltransferases, disruptor of telomeric silencing 1 like (DOT1L) and protein arginine methyltransferase 5 (PRMT5), revealed even more pronounced effects. In summary, we uncovered MAT2A as a key regulator in *MLL* leukemogenesis and its inhibition led to significant anti-leukemic effects. Therefore, our study paves the avenue for clinical application of PF-9366 to improve the treatment of poor prognosis *MLL*r leukemia.

## 1. Introduction

Rearrangements of the *mixed-lineage leukemia (MLL)* or *lysine methyltransferase 2A (KMT2A)* gene with over 100 known partner genes like *ALL1-fused gene from chromosome (AF) 4* or *AF9* are frequently found in leukemia with a dismal prognosis [1]. Treatment failure and therapy-resistant leukemic clones after conventional chemotherapy are the main cause for relapse and mortality [2]. Thus, uncovering the pathogenic mechanisms that maintain *MLL*-rearranged *(MLL*r) leukemogenesis is crucial to develop new targeted therapies. For this purpose, reliable human models are urgently needed as a platform to study new drugs on their way to clinical translation [3]. Although primary patient cells are presumably the best source to predict responses to specific drugs, they are limited in their application for pharmacological studies due to rapid differentiation and apoptosis in in vitro culture systems [4,5]. Recently, we developed representative human *MLL*r models using CRISPR/Cas9 based on patient-specific sequences with complete translocation between the *MLL* and *AF4* or *AF9* gene modeling the consequences of endogenous oncogene activation and hereby mimicking the patient’s disease [6]. In contrast to primary leukemic cells leading to rapid differentiation in in vitro cultures, our CRISPR/Cas9-*MLL*r model demonstrates unlimited in vitro growth potential amenable to fast and efficiently conduct pharmacological studies with high translational character.

Methionine adenosyltransferase 2A (MAT2A) is a key regulator in cellular metabolism and catalyzes the reaction of L-methionine and adenosine triphosphate (ATP) to S-adenosylmethionine (SAM) [7]. SAM acts as the main methyl donor for many methyltransferases in the cell that are obligatory for many functions like the processing of DNA, RNA, or proteins [8]. MAT2A is overexpressed in various tumors like hepatic cancer, breast cancer, or colon cancer [9,10,11,12]. However, in leukemia, the pathogenic function of MAT2A was only defined within specific leukemia subtypes, such as T- acute lymphoblastic leukemia (ALL) [13,14]. Epigenetic dysregulation plays a pivotal role in *MLL*r leukemogenesis and (hypo)methylation of promoters alters gene expression activity, which is in turn directly dependent on cell metabolism [15,16,17]. Within this epigenetic control center, SAM serves as a substrate for many important methyltransferases like H3K79 methyltransferase disruptor of telomeric silencing 1-like (DOT1L) or protein arginine methyltransferase 5 (PRMT5), maintaining *MLL*r pathogenesis.

Here, we show that MAT2A is aberrantly overexpressed in *MLL*r leukemia compared to non-*MLL*r leukemia and healthy controls. Recently, a new MAT2A inhibitor (MAT2Ai), PF-9366, has been developed, allowing us to further investigate the impact of MAT2A on *MLL*r pathogenesis [18]. We administered MAT2Ai in our *MLL*r leukemia model, in *MLL*r and non-*MLL*r cell lines and revealed anti-leukemic effects like a reduction of cell growth and viability, enhanced differentiation, induction of apoptosis, reduction of histone methylation, and the most pronounced impairment of the cell cycle with the highest sensitivity in *MLL*r cells. Remarkably, the addition of PF-9366 to standard chemotherapy or targeted therapies against PRMT5 or DOT1L, either as pretreatment or co-administered simultaneously, further induced *MLL*r cell death.

In conclusion, our data highlight that MAT2A plays a key role in *MLL*r cell survival and accentuates the therapeutic potential of MAT2A inhibition. We thereby establish the preclinical rationale to target MAT2A in leukemia patients harboring *MLL* translocations.

## 2. Results

### 2.1. Human CRISPR/Cas9-MLLr Model Reveals MAT2A as a Possible Target in MLLr Leukemia

Recently, we reported the generation of a reliable human CRISPR/Cas9-*MLL*r model based on patient sequences under endogenous oncogene expression [6]. This model is convincing by both indefinite growth potential in *in vitro* culture systems and the possibility to identify therapeutically relevant downstream effects of *MLL-AF4* and -*AF9* translocations.

**Figure 1:**
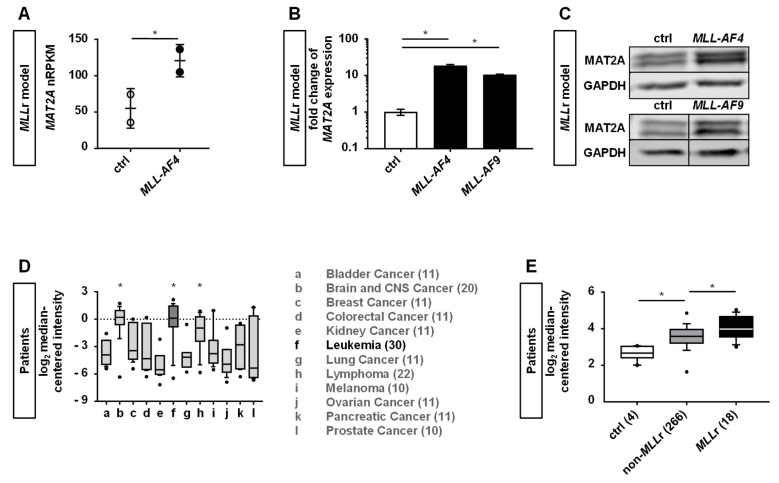
Human CRISPR/Cas9-*MLL*r model reveals MAT2A as a possible target in *MLL*r leukemia. (**A**) RNA sequencing of human CRISPR/Cas9-*MLL-AF4* (two different donors, *n* = 2) compared to the respective controls (ctrl, *n* = 2, CD34^+^ huCB cells nucleofected with Cas9 alone and cultured for the same time) revealed upregulated expression of *MAT2A*. Values in normalized reads per kilobase million (nRPKM), each dot represents a sample. (**B**) Validation of *MAT2A* expression was performed by RT-qPCR. *MLL-AF4* and *MLL-AF9* cells were normalized to culture-expanded CD34^+^ huCB control cells (ctrl). Experiment was performed in biological duplicates (*n* = 2) with horizontal bars representing the mean. Error bars indicate standard deviation (SD). One-way ANOVA was used with Dunnett correction: * *p* < 0.05. (**C**) Representative Western blot analysis shows increased MAT2A expression in *MLL*r cells compared to culture expanded CD34^+^ huCB control cells (factor 1.8 for *MLL-AF4* and factor 1.7 for *MLL-AF9*). Glyceraldehyde-3-phosphate dehydrogenase (GAPDH) was used as the loading control. Blots are cropped and full images are displayed in Figure S1. (**D**) Upregulated *MAT2A* expression in leukemic patient samples compared to other cancer types (data from oncomine.org) [19]. Boxes indicate the range from the 25th through 75th percentiles; the horizontal lines represent the median; error bars indicate the range from 10th through 90th percentiles; the dots show the maximum and minimum values. Student’s t test was used: * *p* < 0.05. (**E**) *MAT2A* expression in *MLL*r leukemia compared to non-*MLL*r leukemia and healthy controls, respectively (GSE28497, data from oncomine.org) [20]. Boxes indicate the range from 25th through 75th percentiles; the horizontal lines represent the median; error bars indicate the range from 10th through 90th percentiles; the dots show the maximum and minimum values. Mann–Whitney *U* test was used: * *p* < 0.05.

Comparing the transcriptome of the human CRISPR/Cas9-*MLL*r cells with respective control cells (CD34^+^ human cord blood (huCB) cells nucleofected with Cas9), we found MAT2A significantly overexpressed in our CRISPR/Cas9-*MLL*r model, suggesting a potential central role in this disease (Figure 1A). To further confirm the RNA-seq results, we performed RT-qPCR and Western blot analyses and again revealed higher levels of *MAT2A* in the *MLL-AF4* and *-AF9* translocated cells compared to the respective control cells (Figure 1B,C; Figure S1). To generally elucidate the impact of MAT2A in cancer, we mined the literature and compared the expression level of *MAT2A* in different patient cancer entities. Strikingly, we discovered the highest levels of *MAT2A* expression in brain cancer, leukemia, and lymphoma (Figure 1D) [19]. In primary patient leukemia, *MLL*r leukemia showed the highest *MAT2A* expression compared to non-*MLL*r leukemia and healthy controls, respectively (Figure 1E) [20].

These data indicate that MAT2A plays a pivotal role in *MLL*r leukemogenesis, amenable as a potential target in the treatment of *MLL*r leukemia.

### 2.2. MAT2A Inhibition Impairs Proliferation and Viability of MLLr Cells and Is Associated with Decreased Global Histone Methylation

**Figure 2:**
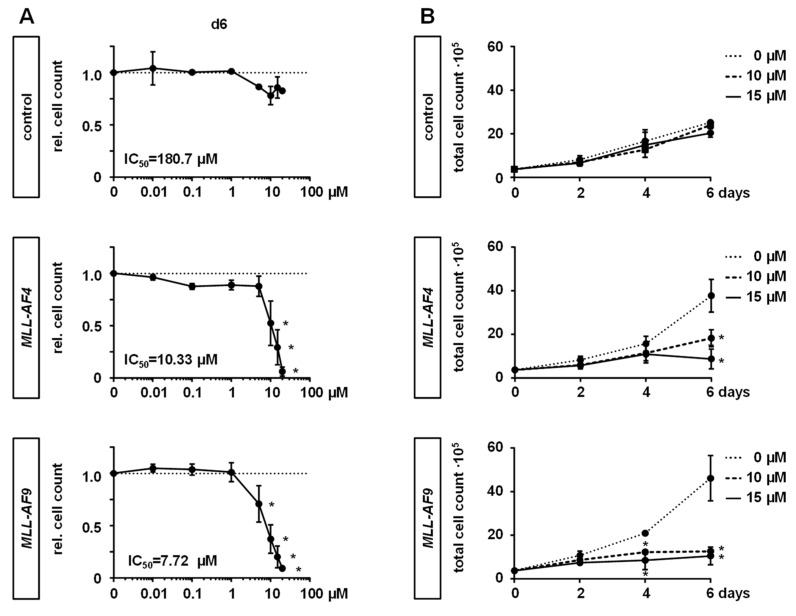

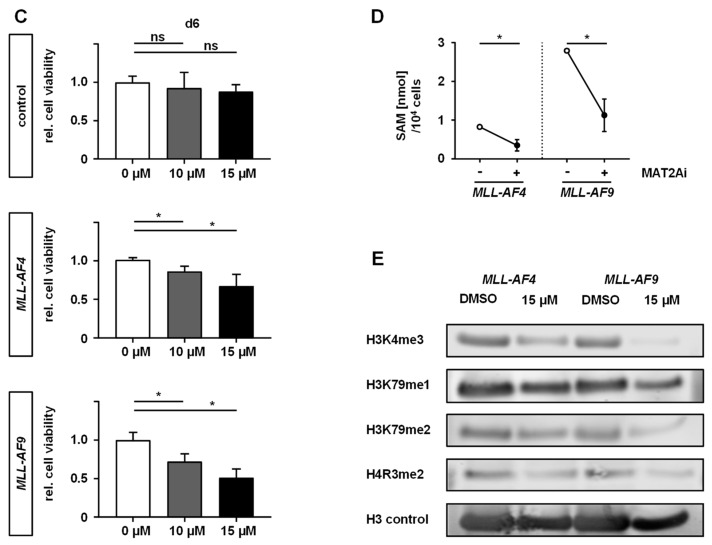
MAT2A inhibition impairs the proliferation and viability of *MLL*r cells, resulting in reduced SAM levels and histone methylation. (**A**) *MLL-AF4/-AF9* or culture-expanded CD34^+^ huCB control cells were treated with increasing concentrations of PF-9366 or vehicle (DMSO) for 6 days and the relative cell count was determined by flow cytometry using counting beads. Pooled data of three biological replicates (*n* = 3) performed in technical triplicates are shown. IC_50_ values of the dose–response curves were interpolated from a four-parameter logistic model constrained to 0 and 1 in GraphPad Prism. Dots represent the mean. Error bars indicate SD. One-way ANOVA was used with Dunnett correction: *, *p* < 0.05. (**B**) Proliferation curves were assessed by treating the indicated cells with PF-9366 (10, 15 µM) or control (DMSO) and counting cells following Trypan blue staining every second day. The mean of pooled data of three biological replicates (*n* = 3) performed in technical triplicates is shown. Dots represent the mean. Error bars indicate SD. One-way ANOVA was used with Dunnett correction: *, *p* < 0.05. (**C**) Cell viability of *MLL*r and control cells upon inhibition treatment with PF-9366 (10, 15 µM) or control (DMSO) was determined after 6 days by fluorescence-based read of alamarBlue assay. Experiment was performed in three biological replicates (*n* = 3) and technical triplicates. Bars represent the mean. Error bars indicate SD. One-way ANOVA was used with Dunnett correction: *, *p* < 0.05. (**D**) *MLL*r cells were treated with 15 µM PF-9366 or DMSO as a control for 6 days. Intracellular SAM levels of 10^4^ cells were quantified by fluorescence-based Bridge-It Assay Platform Technology using a SAM standard curve. Results of two independent experiments (*n* = 2) are displayed. Student’s *t*-test was used: *, *p* < 0.05. (**E**) Western blot analyses of extracted histones from *MLL*r cells treated with 15 µM or DMSO as the control for 6 days are displayed showing decreased global methylation of H3K4me3, H3K79me1, H3K79me2, and H4R3me2. Blots are cropped and full images are displayed in Figure S4.

Recently, PF-9366, a new allosteric inhibitor of MAT2A, has been developed, allowing us to study the impact of MAT2A inhibition on *MLL*r leukemogenesis by using our CRISPR/Cas9-*MLL-AF4/-AF9* models and *MLL*r cell lines SEM *t*(4;11) and THP-1 *t*(9;11) in contrast to the non-*MLL*r cell line SKM-1 [18]. To define the toxicity and potential side effects of the treatment, healthy cells were utilized as controls. First, we measured the IC_50_ values of each cell type by increasing concentrations of PF-9366 and by counting cells following staining with Trypan blue or using counting beads at day 6. For our CRISPR/Cas9-*MLL*r models, we revealed IC_50_ values of around 10 µM (*MLL-AF4*: 10.33 µM; *MLL-AF9*: 7.72 µM), whereas control cells were clearly more robust against the inhibitor treatment (Figure 2A). *MLL*r cell lines were most susceptible against MAT2A inhibition (SEM: 3.815 µM or 3.146 µM; THP-1: 4.210 µM or 5.334 µM) whereas the non-*MLL*r cell line SKM-1 was less affected (12.75 µM or 10.72 µM) (Figure S2A,B). According to these results, we considered concentrations of 10 and 15 µM in further experiments. For the assessment of proliferation, we treated the cells again with PF-9366 (10 and 15 µM or dimethyl sulfoxide (DMSO) as the control) and monitored the proliferation of CRISPR/Cas9-*MLL*r or control cells, THP-1, SEM, or SKM-1, by counting the cell numbers every other day. We observed significantly reduced proliferation of CRISPR/Cas9-*MLL*r cells and *MLL*r cell lines, while this treatment had no or only little effect on control cells or SKM-1, respectively (Figure 2B; Figure S2C). Likewise, following 6 days of inhibitor treatment, the viability was significantly reduced in CRISPR/Cas9-*MLL*r cells and *MLL*r cell lines, whereas there was only a modest impact on control cells or SKM-1 (Figure 2C; Figure S2D). We could further confirm these findings by a *MAT2A* knockdown using small interfering (si) RNA in Jurkat, THP-1, and CRISPR/Cas9-*MLL*r cells, leading to both a reduction of proliferation and an increase of apoptosis, especially in *MLL*r cells (Figure S3A–C). To test whether the MAT2A inhibition results in a consequent reduction of the MAT2A product SAM, we determined the intracellular SAM levels upon treatment and could confirm a successful decrease of the SAM concentration (Figure 2D).

Finally, we evaluated the histone modifications in our CRISPR/Cas9-*MLL*r models upon MAT2A inhibition and revealed reductions of H3K4me3 (to 37% and 9%), H3K79me1 (to 68% and 63%), H3K79me2 (to 58% and 29%), and H4R3me2 (to 46% and 38%) (Figure 2E, Figure S4).

These data implicate that MAT2A inhibition leads to a specific anti-leukemic effect by a reduction of proliferation and viability and by suppression of global histone methylation in *MLL*r leukemia.

### 2.3. Inhibition of MAT2A Restrains Cell Cycle and Forces Maturation and Apoptosis of MLLr Cells

**Figure 3:**
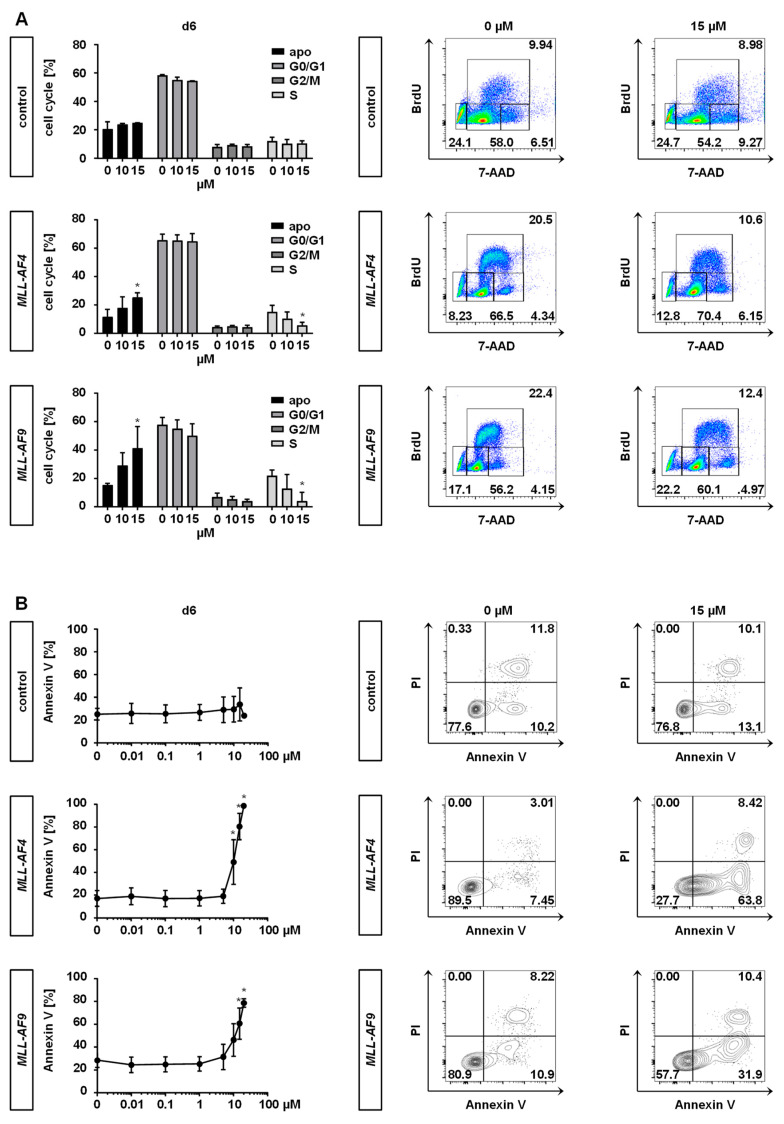
Inhibition of MAT2A reduces DNA synthesis and induces apoptosis of *MLL*r cells. *MLL-AF4/-AF9* or culture-expanded CD34^+^ huCB control cells were treated with PF-9366 (10 or 15 µM) or vehicle (DMSO) for 6 days. (**A**) Pooled data (left) and representative (right) cell cycle analyses of single cells from three biological replicates (*n* = 3) performed in technical triplicates are shown. Data were acquired using BrdU staining and flow cytometry. Bars represent the mean. Error bars indicate SD. One-way ANOVA was used with Dunnett correction: *, *p* < 0.05. (**B**) Annexin V staining revealed translocation of phosphatidylserine to the outer leaflet of the plasma membrane in apoptotic cells. Pooled (left) and representative (right) data from three biological replicates (*n* = 3) performed in technical triplicates are shown. Dots represent the mean. Error bars indicate SD. One-way ANOVA was used with Dunnett correction: *, *p* < 0.05.

To further investigate the consequences of MAT2A inhibition on *MLL*r cells, we performed cell cycle analysis. Bromodeoxyuridine (BrdU) and 7-amino-actinomycin D (7-AAD) staining led to both a decrease in the percentage of CRISPR/Cas9-*MLL*r cells in the S phase and an increase of apoptotic cells, whereas control cells were not affected (Figure 3A). These findings were confirmed by Annexin V staining upon PF-9366 treatment in a dose-dependent manner using flow cytometry (Figure 3B). Similar to our *MLL*r models, we detected a strong induction of apoptosis as well as a reduction of DNA synthesis in *MLL*r cell lines, while the non-*MLL*r cell line SKM-1 was only affected mildly. This indicates a specific dependency of *MLL*r leukemogenesis on MAT2A, whereas SKM-1 cells unfold their pathogenicity by an alternative pathway (Figure S5).

**Figure 4:**
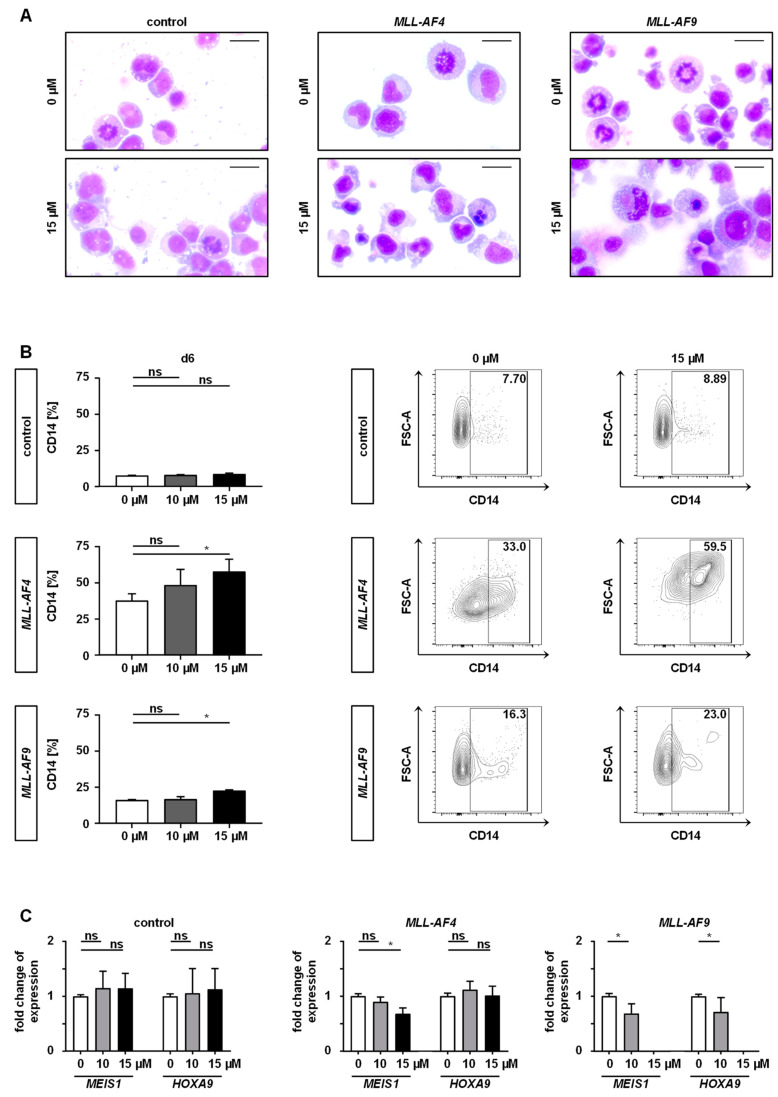
MAT2A inhibition increases differentiation and diminishes *MLL*r-specific target gene expression. *MLL-AF4/-AF9* or culture-expanded CD34^+^ huCB control cells were treated with 10 or 15 µM of PF-93 66 or vehicle (DMSO) for 6 days. (**A**) Representative morphologies of *MLL*r and control cells are shown displaying increased cell differentiation and cell death after drug treatment. Scale bars define 20 µm. (**B**) Pooled (left) and representative (right) CD14 expression of living single cells from three biological replicates (*n* = 3) performed in technical triplicates measured by flow cytometry are shown. Bars represent the mean. Error bars indicate SD. One-way ANOVA was used with Dunnett correction: *, *p* < 0.05. (**C**) Target gene expression was analyzed by RT-qPCR demonstrating decreasing levels of *MEIS1* and *HOXA9* upon inhibition with PF-9366 (10 or 15 µM). Experiment was performed in three biological replicates (*n* = 3) and technical triplicates. Bars represent the mean. Error bars indicate SD. One-way ANOVA was used with Dunnett correction: *, *p* < 0.05. Student’s *t* test was used for *MLL-AF9*: *, *p* < 0.05.

One specific characteristic of *MLL*r leukemia is the expression of elevated target genes, hereby preserving immaturity [21,22,23]. Therefore, we investigated the expression of *MLL*r target genes and differentiation upon inhibition with PF-9366. For maturation analysis, we performed May-Gruenwald-Giemsa staining to evaluate cell morphology and additionally determined CD14 as a marker of differentiation by flow cytometry following 6 days of PF-9366 treatment. Untreated (DMSO control) CRISPR/Cas9-*MLL*r cells presented an immature morphology whereas treatment with PF-9366 resulted in macrophage-like cells with an increase of apoptotic cells consistent with the upregulation of CD14 expression (Figure 4A,B). Consistently, a trend to downregulate the target gene expression of *MEIS1* and *HOXA9* upon PF-9366 treatment was observed, irrespective of day 4 or 6, although significance was not reached in all performed experiments (Figure 4C). These data suggest that the inhibition of MAT2A results in cell differentiation, induction of cell cycle arrest, and finally apoptosis in MLL fusion protein-driven leukemogenesis without any impact on control cells.

### 2.4. Downstream Effects of MAT2A Inhibition on Gene Expression

As a next step, we performed RNA-seq of CRISPR/Cas9-*MLL*r cells treated with PF-9366 compared to DMSO treatment after 4 days to unravel the consequences of MAT2A inhibition on genome-wide expression.

**Figure 5:**
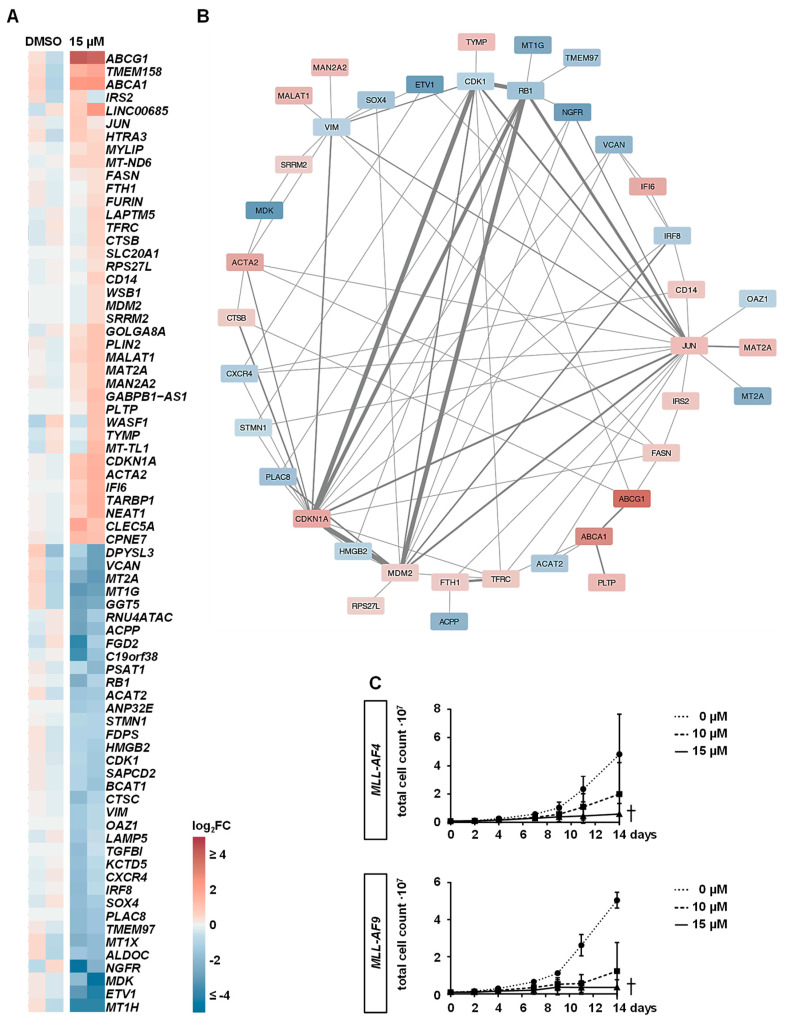
Downstream effects of MAT2A inhibition on gene expression. (**A**) *MLL-AF4* cells were treated with 15 µM of PF-9366 or vehicle (DMSO) for 4 days and two biological replicates (*n* = 2) were used for RNA-seq. DEGs are displayed with 38 genes upregulated and 36 genes downregulated. The interactome of all DEGs is shown in (**B**) with the main effect of MAT2A inhibition on cell cycle progression notable by the interacting genes *CDKN1A/p21*, *CDK1*, *RB1*, and *MDM2.* (**C**) For long-term experiments, *MLL-AF4/-AF9* cells were treated with 10 or 15 µM of PF-9366 or vehicle (DMSO) for a minimum of 2 weeks. Stable inhibition of proliferation is shown by two biological replicates (*n* = 2) performed in technical triplicates.

RNA-seq analysis revealed that upon MAT2A inhibition, only a selection of 74 genes were differentially expressed, with 38 up- and 36 downregulated (Figure 5A; Table S2). To further validate our RNA-seq results, we performed RT-qPCR of some genes (Figure S6). We were able to retrieve our previous functional results concerning cell cycle, proliferation, and maturation block as important hallmarks of leukemogenesis and could further identify altered pathways belonging to the cholesterol and lipid metabolism. First, the most frequently affected genes were those controlling G1/S cell cycle progression (e.g., *CDKN1A/p21*, *CDK1*, *RB1*, and *MDM2*) as shown in the center of the interactome of the 74 differentially expressed genes (DEGs) (Figure 5B). Particularly, CDKN1A as cell cycle inhibitor p21 has already been shown to play a key role in the context of *MLL*r leukemic stemness, cell cycle, proliferation, and immaturity, acting as a biologically important target in *MLL*r leukemogenesis [24,25,26]. Furthermore, several genes influencing cell maturity showed altered expression, such as the classical marker of differentiation, *CD14,* which was upregulated upon PF-9366 treatment [27]. Similarly, both a decrease of *SOX4* and *PLAC8* expression and an increase of the long non-coding RNAs *NEAT1* and *MALAT1* additionally interrupt the maturation block, leading to myeloid differentiation of AML cells [28,29,30,31]. Importantly, similar to our previous RT-qPCR results, downregulation of *MLL*r target genes did not reach significance, and were not among the 74 DEGs. Hence, MAT2A seems to additionally drive its pathogenic course by alternative pathways [32]. Interestingly, upon MAT2A inhibition, we detected the cholesterol efflux transporters *ABCG1* and *ABCA1* of the cholesterol metabolism to be significantly upregulated, resulting in known anti-proliferative and apoptotic effects in hematological diseases [33,34]. Another gene of this cholesterol group, presenting with reduced expression upon PF-9366 treatment, was *TMEM97* [35]. It is known that overexpression of *TMEM97* is positively correlated with tumor proliferation, metastasis, and reduced survival in various cancer types. Therefore, first efforts have already been made to use it as a target in cancer treatment [36,37,38].

Finally, two well-known leukemogenic targets, *LAMP5* and *CXCR4*, were downregulated upon PF-9366 treatment. *LAMP5* is specifically and highly expressed in *MLL*r leukemia. Recently, it has been shown that LAMP5 acts as a direct target of DOT1L resulting in autophagic suppression and protection of *MLL* fusion proteins [39]. CXCR4 together with CXCL12 is important for the interaction of leukemic stem cells (LSCs) with the microenvironment by promoting quiescence and inhibiting apoptosis in LSCs and therefore inducing therapeutic resistance in leukemia [40,41].

In addition, we detected a compensatory upregulation of *MAT2A* upon inhibition (Figure 5A) as shown before [18]. To exclude the development of the drug resistance of the *MLL*r cells, we performed long-term experiments with the PF-9366-treated CRISPR/Cas9-*MLL*r cells and revealed a persistent inhibition of proliferation despite the upregulation of *MAT2A* on the mRNA level (Figure 5C).

Taken together, our data show that MAT2A is required for the survival advantage and the myeloid differentiation block as characteristics of *MLL*r leukemia.

### 2.5. PF-9366 Sensitizes MLLr Cells to Chemotherapy and Acts Synergistically in Combination with Targeted Therapies

**Figure 6:**
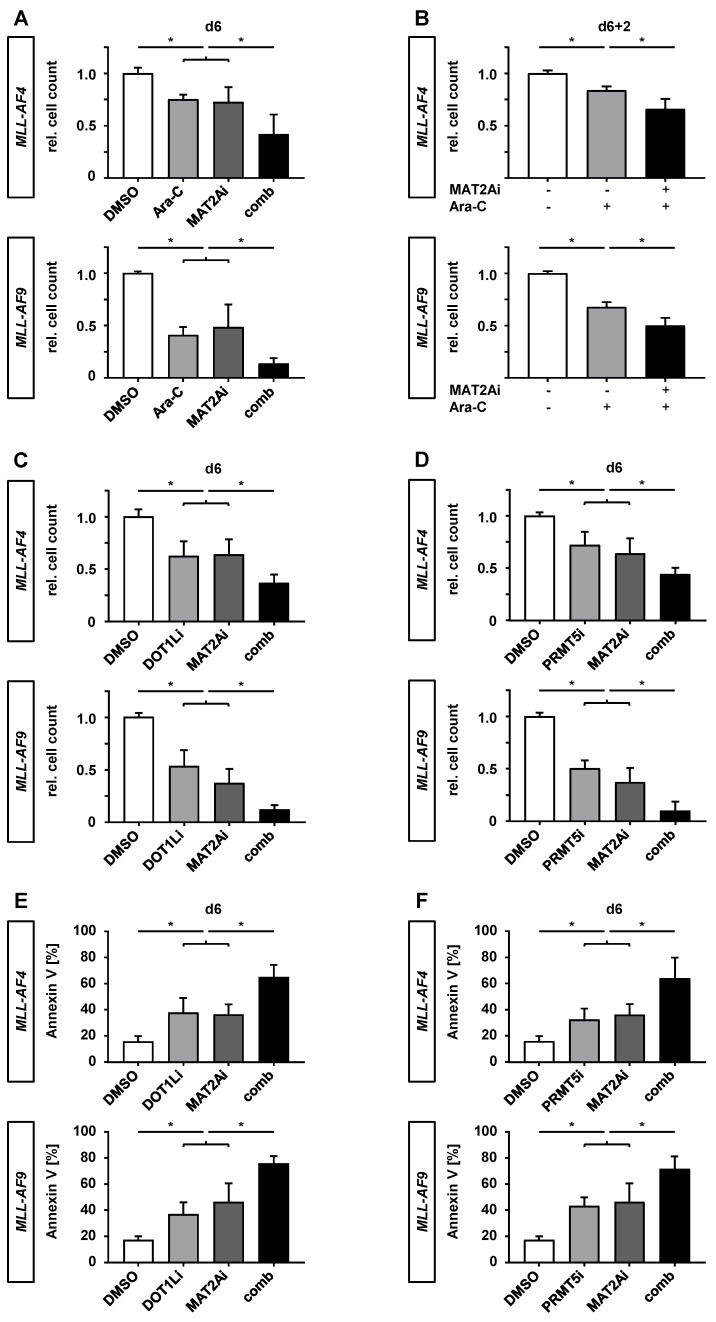
PF-9366 sensitizes *MLL*r cells to chemotherapy and acts synergistically in combination with targeted therapies. (**A**) *MLL-AF4/-AF9* cells were treated with 10 nM cytarabine (Ara-C) and 10 µM PF-9366 (MAT2Ai) simultaneously for 6 days. Single treatments or vehicle (DMSO) were used as controls, respectively. Experiment was performed in three biological replicates (*n* = 3) and technical triplicates. (**B**) Initial treatment with PF-9366 (MAT2Ai +) or vehicle (MAT2Ai −, DMSO) for 6 days was washed out and cells were reseeded with 10 nM cytarabine (Ara-C +) or vehicle (Ara-C −, PBS) and monitored for 2 days (6 + 2). Experiment was performed in three biological replicates (*n* = 3) and technical triplicates. Relative cell count was determined by flow cytometry using counting beads. (**C**,**D**) *MLL-AF4/-AF9* cells were treated with either 1 µM EPZ004777 (DOT1Li), 1 µM EPZ015666 (PRMT5i), or 10 µM PF-9366 (MAT2Ai), in respective combinations or vehicle (DMSO) for 6 days. Relative cell count was analyzed by flow cytometry using counting beads. Pooled data of three biological replicates (*n* = 3) performed in technical triplicates are shown. (**E**,**F**) Annexin V staining revealed apoptotic cells in *MLL-AF4/-AF9* cells treated with the indicated compounds for 6 days. Experiment was performed in three biological replicates (*n* = 3) and technical triplicates. Data were acquired using flow cytometry. Bars represent the mean. Error bars indicate SD. One-way ANOVA was used with Sidak correction: *, *p* < 0.05.

We further investigated the anti-leukemic effect of PF-9366 with cytarabine, one of the most used chemotherapeutic agents to treat patients with AML, in combined and sequential experiments to evaluate the function of PF-9366 as a potential co-medication. First, we determined the IC_50_ of cytarabine by adding increasing concentrations to the human CRISPR/Cas9-*MLL*r cells (IC_50_ = 9.04 nM; Figure S7A). Accordingly, a concentration of 10 nM cytarabine was used in the following experiments. After a period of 6 days, the simultaneous application of cytarabine and PF-9366 induced a superior anti-leukemic effect compared to respective agents alone determined with counting beads by using flow cytometry (Figure 6A). For the sequential treatment experiments, we pretreated CRISPR/Cas9-*MLL*r cells with PF-9366 or DMSO for 6 days, washed them out, reseeded equal numbers of cells, and added cytarabine to the culture for another 2 days (Figure 6B). Remarkably, although the MAT2Ai was removed from the culture, pretreated CRISPR/Cas9-*MLL*r cells were more affected by cytarabine than untreated cells, indicating a lasting impact of MAT2A inhibition on the methylation status of CRISPR/Cas9-*MLL*r cells.

Recently, DOT1L and PRMT5 have been identified as suitable targets with only minor side effects for the treatment of poor prognosis *MLL*r leukemia and we could demonstrate that the combination of both resulted in synergistic effects [6,26,42]. As MAT2A synthesizes SAM as a key methyl donor for both methyltransferases, we expected an additional enhancement of the PF-9366-induced anti-leukemic effect by combining the inhibitors (Figure S7B), aiming to overcome the only moderate response on *MEIS1* and *HOXA9* expression. First, we determined the dose–response curves of single and combination treatments of both inhibitor variants (MAT2Ai + DOT1Li and MAT2Ai + PRMT5i) in CRISPR/Cas9-*MLL*r cells at a constant ratio of equipotency of 1:10 using counting beads and flow cytometry (Figure S7C,D). For synergy determination, IC_50_ values were interpolated and isobolograms at the 50% effect level as well as the calculation of combination indices (CI < 1) indicated synergism (Figure S7E,F). Likewise, CRISPR/Cas9-*MLL*r cells showed a significantly reduced cell count after 6 days (Figure 6C,D) and a higher percentage of apoptotic cells in combinational treated cells with MAT2Ai and DOT1Li or PRMT5i compared to single agents (Figure 6E,F). The anti-leukemic effects of the different therapeutic strategies could be confirmed by using *MLL*r cell lines SEM and THP-1, while the non-*MLL*r cell line SKM-1 showed only a moderate response (Figure S8).

Importantly, neither the combination of PF-9366 with chemotherapy nor with targeted therapies had any negative impact on healthy control cells (Figure S9).

Taken together, these data provide evidence for the beneficial combinational treatment of PF-9366 with both chemotherapy and well-tolerated targeted therapies against DOT1L and PRMT5 participating in the MAT2A pathway.

## 3. Discussion

In this study, we demonstrated an MAT2A dependency in *MLL*r leukemogenesis, constituting an ideal target in the treatment of poor prognosis *MLL*r leukemia. We used our recently developed human CRISPR/Cas9-*MLL*r model based on complete translocations of the *MLL* and *AF4* or *AF9* gene, respectively. These genome-edited cells recapitulate all molecular aspects of the disease and promote unlimited cell growth in vitro, serving as an ideal human model to uncover pathogenic pathways and consequently to test potential new drugs fast and efficiently for clinical translation.

*MLL*r leukemia unfold their leukemogenic potential by epigenetic dysregulations, leading to alterations of the cellular metabolism and hereby promoting leukemic cell proliferation and immaturity [15,21]. In this context, SAM, as a product of MAT2A, is the major cellular methyl donor regulating gene expression by epigenetic mechanisms as well as proliferation, apoptosis, and differentiation [8]. Interestingly, it has already been shown that the nucleoside analogon clofarabine, leading to depletion of SAM, promoted anti-leukemic effects in *MLL*r leukemia [43]. Due to the recent lack of appropriate MAT2A inhibitors, cycloleucine as a substrate-competitive inhibitor with low affinity to MAT2A, has been used to block MAT2A non-specifically in T-cell leukemia and other tumor entities with unclear effects and also affecting other essential pathways [13,44]. Moreover, the usage of cycloleucine as an anti-cancer drug can result in severe side effects, most notably central nervous system (CNS) toxicity. Thus, cycloleucine is not suitable to interpret the therapeutic efficacy of MAT2A inhibition [45].

Recently, the MAT2A inhibitor PF-9366 with high affinity has been described to efficiently block SAM synthesis, allowing for the first time an evaluation of MAT2A as a therapeutic target in *MLL*r leukemia [18]. In the meantime, AG-270, another MAT2A inhibitor with high affinity, has been developed that is already undergoing a phase I clinical trial in patients suffering from lymphoma or solid tumors (NCT03435250, clinical trials.gov), supporting the rationale to target MAT2A in leukemia. However, the effect on proliferation upon increased SAM levels is highly dependent on the cell type, with only low proliferation activities in hepatocytes whereas lymphocytes responded with increased proliferation. Therefore, detailed analysis on leukemic cells is crucial to understand the SAM-dependent cellular metabolism in this specific cancer subtype [46,47].

We analyzed both publicly available datasets and our own data and retrieved MAT2A as a major player in *MLL*r leukemogenesis, showing the highest expression level within this specific leukemia subtype. Moreover, by comparing the response to MAT2A inhibition in *MLL*r- versus non-*MLL*r cell lines, we could identify *MLL*r cell lines as being most susceptible to PF-9366 treatment, indicating a high dependency on MAT2A in the *MLL*r pathogenicity. Importantly, during polyamine biosynthesis, methylthioadenosine (MTA) occurs as a by-product of elevated SAM levels, which in turn can lead to inhibition of H3K4 methylation, further preventing the physiological cell function of MLL [48,49].

Our CRISPR/Cas9-*MLL*r model with an indefinite growth potential in in vitro cultures provided robust data about the inhibition of MAT2A leading to reduced proliferation, impairment of cell cycle, maturation, suppression of histone methylation, and finally apoptosis of the leukemic cells. To validate the anti-leukemic effects of PF-9366, we performed knockdown experiments of MAT2A by using siRNAs. Although, the achieved result by using siRNA was smaller compared to the inhibitor treatment due to the transient and partial downregulation of MAT2A, we could confirm our findings and provided the therapeutic rationality of blocking MAT2A by PF-9366. Comparing the gene expression profiles before and after PF-9366 treatment, we could identify DEGs involved in proliferation, maturation, and most pronounced in metabolism of the cell cycle with CDKN1A as a pivotal regulator, confirming our functional experiments obtained in the in vitro culture systems. Interestingly, genes coding for cholesterol efflux transporters, such as *ABCG1* and *ABCA1*, with known functions as proliferative inhibitors of malignant cells were disturbed [50]. It is known that the cholesterol and lipid metabolism with high metabolic rates, leading to wasting syndromes, plays a critical role in many neoplasms like myeloproliferative disorders.

In lung cancer, Wang et al. showed that tumor-initiating cells, the presumably most relevant cell type to target within the tumor bulk, possess high MAT2A-driven methionine cycle activity, leading to epigenetic changes and tumor initiation [51]. In line with our study, this observation further supports the relevance of MAT2A in *MLL*r leukemia. In our model, further experiments using different subgroups expressing CD34 or CD38 are needed to determine the metabolic dependency of methionine in *MLL*r leukemia-initiating cells.

In *MLL*r leukemia, the MAT2A product SAM serves as a substrate for many important methyltransferases like the H3K79 histone methyltransferase DOT1L or PRMT5, both involved in the pathogenesis of the disease. This provides the rationale to further enhance the anti-leukemic effect of MAT2A inhibition by additionally blocking these methlytransferases [6,26,52,53]. To enhance the cytotoxic effects by disturbing the metabolic cycle based on SAM, we performed combinational treatments with the respective inhibitors of DOT1L or PRMT5 and MAT2A. Strikingly, we could demonstrate synergistic anti-leukemic effects, serving as a foundation of a reasonable therapeutic combinational strategy with minimal side effects in clinical practice.

Taken together, our results provide a proof-of-principle and preclinical evidence that MAT2A inhibition leads to anti-leukemic effects in *MLL*r leukemia and can easily be combined with other targeted therapies or chemotherapy to overcome the poor prognosis in *MLL*r leukemia.

## 4. Materials and Methods

### 4.1. Human CRISPR/Cas9-MLLr Model

CD34^+^ hematopoietic stem and progenitor cells (HSPCs) were isolated from fresh human umbilical cord blood (huCB) obtained from the Center for Women’s Health (Department of Gynecology) of the University Hospital Tuebingen (IRB approval 751/2015BO2) and maintained in culture as previously described [6]. Written consent was obtained from all patients in compliance with the Declaration of Helsinki. CRISPR/Cas9 was used to target patient-specific *MLL-AF4* and *-AF9* breakpoints for *MLL*r model induction as previously described [6,54,55,56].

### 4.2. Cell Culture

SKM-1 cells (DSMZ ACC 547, Braunschweig, Germany) were cultured in Roswell Park Memorial Institute (RPMI) 1640 medium (Gibco, Thermo Fisher Scientific, Waltham, MA, USA) with 15% filtered fetal bovine serum (FBS) (Merck-Millipore, Darmstadt, Germany) supplemented with 1% penicillin/streptomycin (P/S) (Lonza, Basel, Switzerland). SEM cells (DSMZ ACC-546) were cultured in Iscove’s modified Dulbecco’s medium (IMDM, Lonza) with 10% filtered FBS (Merck-Millipore) supplemented with 1% P/S (Lonza). THP-1 cells (ATCC TIB-202, Manassas, VA, USA) were cultured in RPMI 1640 with 10% filtered FBS (Merck-Millipore) supplemented with 1% P/S (Lonza). All cell lines recently tested negative for mycoplasma contamination (PCR Mycoplasmen - Testkit II, AppliChem GmbH, Darmstadt, Germany).

### 4.3. Quantitative Reverse Transcriptase-PCR (RT-qPCR)

Total RNA was isolated with the NucleoSpin RNA Kit (Macherey Nagel, Dueren, Germany) and cDNA was generated using RevertAid H Minus Reverse Transcriptase, RiboLock RNase Inhibitor, dNTP Mix, and Random Hexamers (all Thermo Fisher Scientific) according to the manufacturer’s protocol. RT-qPCR was performed for the detection of *MAT2A*, *MEIS1*, and *HOXA9* or *CDKN1A*, *LAMP5*, *CXCR4*, and *CDK1* by Maxima SYBR Green qPCR Master Mix (Thermo Fisher Scientific) according to the manufacturer’s protocol and primers listed in Table S1 (Sigma-Aldrich, St. Louis, MO, USA). As a housekeeper, 18S rRNA was amplified using the 18S rRNA probe [JOE]TGCTGGCACCAGACTTGCCCTC[TAM] and Maxima Probe qPCR Master Mix (Thermo Fisher Scientific). Relative RT-qPCR analysis was performed in a LightCycler 480 Instrument II (Roche Life Science, Penzberg, Germany) employing the ddCT method. The results were normalized on 18S rRNA and respective control cells were used as a calibrator.

### 4.4. Western Blot

For MAT2A protein quantification, cells were harvested and lysed using RIPA buffer (1 % NP40, 0.5 % DOC, 0.1 % SDS, 50 mM TRIS pH 8.0, 80 mM NaCl, 50 mM NaF, 20 mM Na_4_P_2_O_7_, 1 mM EDTA, 1 mM EGTA, and 1 Complete tablets Mini Easypack (Roche) for 10 mL). An equal protein amount was loaded on a 10% SDS gel with Laemmli buffer and PageRuler Plus Prestained Protein Ladder, 10 to 250 kDa (Thermo Fisher Scientific), and blotted on an Immobilon-FL PVDF membrane (Merck-Millipore). After blocking, primary antibodies (GAPDH, clone 14C10 from Cell Signaling, Danvers, MA, USA and MAT2A, polyclonal from Novus Biologicals, Centennial, CO, USA) and the secondary antibody (anti-rabbit IgG, IRDye 680RD, polyclonal from Li-Cor Biosciences, Lincoln, NE, USA) were used. Analysis was carried out at the Li-Cor Odyssey CLx using the Image Studio Software (both Li-Cor Biosciences) for documentation. Relative quantification was performed using glycerinaldehyde-3-phosphate-dehydrogenase (GAPDH) as the reference protein.

### 4.5. Compound Inhibition Assays

MAT2A inhibitor PF-9366 (Carbosynth, Compton, UK), DOT1L inhibitor EPZ004777 (Tocris, Bristol, UK), and PRMT5 inhibitor EPZ015666 (Sigma-Aldrich) were prepared in stock solutions with DMSO [18,57,58,59]. Cytarabine (Ara-C, Stadapharm, Bad Vilbel, Germany) was diluted in PBS for stock solutions. *MLL*r cells and culture-expanded CD34^+^ huCB control cells seeded with 7.5 × 10^5^ cells/mL were subjected to inhibitor treatment in StemMACS HSC Expansion Media XF (Miltenyi, Bergisch Gladbach, Germany) supplemented with 1% penicillin-streptomycin (Lonza), 10% FBS, and 50ng/mL G-CSF, TPO, SCF. FLT3L, IL-3, and IL-6 (Peprotech, Rocky Hill, NJ, USA), and 0.75 µM SR-1 and UM-729 (Stem Cell Technologies, Vancouver, BC, Canada) for a total of 6 days or otherwise as indicated. Cells were retreated and reseeded at the original density every second day. For long-term experiments, treatment was prolonged to a maximum of 2 weeks. For sequential experiments, cells were preincubated for 6 days, compound was washed out, and cells were reseeded at 7.5 × 10^5^ cells/mL followed by respective treatment.

SKM-1 and SEM cells were seeded at 0.5 × 10^6^/mL and THP-1 cells at 0.2 × 10^6^/mL. Cells were subjected to inhibitor treatment for 2, 4, or 6 days in the respective medium described above and reseeded at the original density after 4 days.

### 4.6. Flow Cytometry- and Microscopy-Based Determination of Cell Counts

During compound treatment over 6 days, cell counts for titration experiments and proliferation curves were determined by staining with Trypan blue (Gibco, Thermo Fisher Scientific) using the Neubauer counting chamber. Further, cell counts were analyzed via flow cytometry by staining dead cells with propidium iodide (PI, BD Biosciences, San Jose, CA, USA) and counting beads (Sigma-Aldrich) as previously described [6].

### 4.7. Viability Assay

To determine cell viability, alamarBlue cell viability reagent (Thermo Fisher Scientific) was used. Blue cell-permeable non-fluorescent resazurin is reduced to red highly fluorescent resorufin upon entering living cells. Changes in viability were detected using a fluorescence-based Synergy HTX Multi-Mode Reader and Gen Version 2.0 Data Analysis Software (both BioTek Instruments, Winooski, VT, USA).

### 4.8. BrdU Cell Cycle and Apoptosis Analyses

BrdU incorporation assay and Annexin V apoptosis staining were performed using the FITC BrdU flow kit (BD Biosciences) and FITC Annexin V Apoptosis Detection Kit I (BD Biosciences) according to the manufacturer’s protocol and as previously described [6].

### 4.9. Analysis of Cell Differentiation

Cytospins were prepared and stained as previously described [60]. Images were collected using a Zeiss Primovert with an x40 objective and the Axiocam 105 color camera using ZEN 3.0 blue edition software (all Carl Zeiss AG, Oberkochen, Germany) at a resolution of 2560 x 1920 pixels. Differentiation marker CD14 was detected using anti-CD14-Alexa Fluor 700 antibody (clone HCD14, BioLegend, San Diego, CA, USA) as previously described [6].

### 4.10. RNA Sequencing

RNA was isolated (Macherey Nagel NucleoSpin RNA Kit) and quality assessment was carried out by NanoDrop (Thermo Fisher Scientific) and Bioanalyzer measurements (Agilent, Santa Clara, CA, USA). Sequencing of the Quantseq 3′ mRNA libraries was done on a NextSeq 500 platform (Illumina Inc., San Diego, CA, USA) at a resolution of ~7mio single-end reads per sample and 75 bp in length.

### 4.11. siRNA Knock-Down Experiments

First, 10 × 10^5^ cells were nucleofected using either SE Cell Line 4D-Nucleofector X Kit S and CL-120 or P3 Primary Cell 4D-Nucleofector X Kit S and EO-100 (both Lonza) for cell lines or *MLL*r cells, respectively. For the 20-µL strips, 45 pmol (600 ng) MAT2A siRNA (sense) 5′GGG UGA UGC UGG UUUNGAC Utt and (antisense) 5′AGU CAA ACC AGC AUC ACC Ctt (Sigma-Aldrich) were used [13]. Non-target siRNA MISSION siRNA Universal Negative Control #1 (Sigma-Aldrich) or no siRNA were used as a control. After nucleofection, cells were seeded in 2 × 200 µL of the respective medium. RT-qPCR for MAT2A was used as proof-of-concept and performed on day 1 and 3 as described above. Cells were counted on day 1, 2, and 3 and the Annexin V assay was performed on day 3 as read-out experiments.

### 4.12. Histone Methylation Analysis

Histones were extracted using the Histone Extraction Kit (Abcam, Cambridge, UK). Protein concentration was quantified by DC Protein Assay (Bio-Rad, Hercules, CA, USA) and 20 µg of protein were loaded on a Bolt 10 % Bis-Tris Plus Gel, 10-Well (Thermo Fisher Scientific) using 4X Bolt LDS sample buffer and 10X Bolt Sample Reducing Agent (both Thermo Fisher Scientific). Electrophoresis war carried out at 200 V for 25–35 min using PageRuler Plus Prestained Protein Ladder, 10 to 250 kDa and Bolt MES SDS Running Buffer (both Thermo Fisher Scientific). Protein was transferred to an Immobilon-FL PVDF membrane (Merck-Millipore) at 50 V for 200 min by using Bolt Transfer Buffer (Thermo Fisher Scientific). After blocking, primary antibodies (Tri-Methyl-Histone H3 (Lys4) from Cell Signaling and Anti-Histone H3 (mono methyl K79) antibody, Anti-Histone H3 (di methyl K79) antibody, Anti-Histone H4 (symmetric di methyl R3) antibody, and Anti-Histone H3 antibody from Abcam), and the secondary antibodies (anti-rabbit IgG, IRDye 680RD, polyclonal and ant-rabbit IgG, IRDye 800CW, polyclonal from Li-Cor Biosciences) were used. Analysis was carried out at the Li-Cor Odyssey CLx using the Image Studio Software (both Li-Cor Biosciences) for documentation. Relative quantification was performed using Histone H3 as the reference protein.

### 4.13. SAM Quantification

The Bridge-It S-Adenosyl Methionine (SAM) Fluorescence Assay Kit (Mediomics, St. Louis, MO, USA) was used to determine intracellular SAM levels. First, 5 × 10^4^ cells were lysed and processed according to the manufacturer’s instructions. Supernatants were analyzed and quantified in parallel to the SAM standard curve on a Spark multimode microplate reader (Tecan, Maennedorf, Switzerland).

### 4.14. Statistical Analyses

Student’s t test or one-way ANOVA was used for statistical analysis as indicated in each figure legend. A suitable post hoc test was used for multiple comparisons (Dunnet or Sidak) and *p* < 0.05 was considered statistically significant. IC_50_ values of the dose–response curves were interpolated from a four-parameter logistic model constrained to 0 and 1. Data were analyzed with Prism 7.03 (GraphPad Software, La Jolla, CA, USA). Synergy was calculated using the Chou–Talalay method [61]. The combination index (CI) was used to define additivity (CI = 1), synergism (CI < 1), and antagonism (CI > 1).

### 4.15. Data Sharing Statement

For original data, please contact corina.schneidawind@med.uni-tuebingen.de. Raw sequencing files are available through GEO under accession number GSE141839. Supplementary information is available with the online version of this article.

## 5. Conclusions

For the first time, by using the novel allosteric MAT2A inhibitor PF-9366, we were able to uncover MAT2A as both a key regulator in *MLL* leukemogenesis and as new therapeutic target in the treatment of poor prognosis *MLL*r leukemia. Inhibition of MAT2A leads to reduced proliferation, impairment of cell cycle, maturation, and finally apoptosis of the *MLL*r leukemic cells. Moreover, a combinational treatment of PF-9366 with chemotherapy or targeted therapies against the SAM-dependent methyltransferases DOT1L and PRMT5 revealed even more pronounced anti-leukemic effects as the basis of a reasonable therapeutic combinational strategy with minimal side effects in clinical use.

## Supplementary Materials

The following are available online at https://www.mdpi.com/2072-6694/12/5/1342/s1 [62,63,64,65,66,67], Supplementary Methods. Table S1. Primer sets for RT-qPCR to quantify relative mRNA expression levels, Table S2. Most differentially expressed genes of *MLL-AF4* cells treated with PF-9366 compared to DMSO control., Figure S1. Expanded data for western blots including molecular weight marker as already shown in Figure 1C., Figure S2. PF-9366 preferentially suppresses proliferation and viability of *MLL*r cell lines. Figure S3. siRNA-mediated *MAT2A* knock-down. Figure S4. Expanded data for western blots including molecular weight marker as already shown in Figure 2D. Figure S5 Inhibition of MAT2A diminishes DNA synthesis and enhances apoptosis of *MLL*r cell lines. Figure S6: Validation of RNA-seq data via RT-qPCR. Figure S7. Combinational treatment with PF-9366 and chemotherapy or targeted therapy augments anti-leukemic effects. Figure S8. PF-9366 in combination with chemotherapy or targeted therapies leads to an *MLL*r-specific additive effect validated with cell lines. Figure S9. PF-9366 alone or in combination with targeted therapies has no negative impact on control cells.

## References

[1] Meyer C., Burmeister T., Groger D., Tsaur G., Fechina L., Renneville A., Sutton R., Venn N.C., Emerenciano M., Pombo-de-Oliveira M.S. (2018). The MLL recombinome of acute leukemias in 2017. Leukemia.

[2] Gaussmann A., Wenger T., Eberle I., Bursen A., Bracharz S., Herr I., Dingermann T., Marschalek R. (2007). Combined effects of the two reciprocal t(4;11) fusion proteins MLL.AF4 and AF4.MLL confer resistance to apoptosis, cell cycling capacity and growth transformation. Oncogene.

[3] Kerbel R.S. (2003). Human tumor xenografts as predictive preclinical models for anticancer drug activity in humans: Better than commonly perceived-but they can be improved. Cancer Biol. Ther..

[4] Bruserud O., Gjertsen B.T., Foss B., Huang T.S. (2001). New strategies in the treatment of acute myelogenous leukemia (AML): In vitro culture of aml cells—The present use in experimental studies and the possible importance for future therapeutic approaches. Stem Cells.

[5] Manabe A., Coustan-Smith E., Behm F.G., Raimondi S.C., Campana D. (1992). Bone marrow-derived stromal cells prevent apoptotic cell death in B-lineage acute lymphoblastic leukemia. Blood.

[6] Secker K.A., Keppeler H., Duerr-Stoerzer S., Schmid H., Schneidawind D., Hentrich T., Schulze-Hentrich J.M., Mankel B., Fend F., Schneidawind C. (2019). Inhibition of DOT1L and PRMT5 promote synergistic anti-tumor activity in a human MLL leukemia model induced by CRISPR/Cas9. Oncogene.

[7] Bottiglieri T. (2002). S-Adenosyl-L-methionine (SAMe): From the bench to the bedside--molecular basis of a pleiotrophic molecule. Am. J. Clin. Nutr..

[8] Timp W., Feinberg A.P. (2013). Cancer as a dysregulated epigenome allowing cellular growth advantage at the expense of the host. Nat. Rev. Cancer.

[9] Cai J., Sun W.M., Hwang J.J., Stain S.C., Lu S.C. (1996). Changes in S-adenosylmethionine synthetase in human liver cancer: Molecular characterization and significance. Hepatology.

[10] Simile M.M., Peitta G., Tomasi M.L., Brozzetti S., Feo C.F., Porcu A., Cigliano A., Calvisi D.F., Feo F., Pascale R.M. (2019). MicroRNA-203 impacts on the growth, aggressiveness and prognosis of hepatocellular carcinoma by targeting MAT2A and MAT2B genes. Oncotarget.

[11] Xu J., Wu D., Wang S., Wang Z. (2019). MAT2B expression correlates with poor prognosis in triple-negative breast cancer. Cancer Manag. Res..

[12] Chen H., Xia M., Lin M., Yang H., Kuhlenkamp J., Li T., Sodir N.M., Chen Y.H., Josef-Lenz H., Laird P.W. (2007). Role of methionine adenosyltransferase 2A and S-adenosylmethionine in mitogen-induced growth of human colon cancer cells. Gastroenterology.

[13] Jani T.S., Gobejishvili L., Hote P.T., Barve A.S., Joshi-Barve S., Kharebava G., Suttles J., Chen T., McClain C.J., Barve S. (2009). Inhib61ition of methionine adenosyltransferase II induces FasL expression, Fas-DISC formation and caspase-8-dependent apoptotic death in T leukemic cells. Cell Res..

[14] Halim A.B., LeGros H.L., Chamberlin M.E., Geller A., Kotb M. (2001). Distinct patterns of protein binding to the MAT2A promoter in normal and leukemic T cells. Biochim. Biophys. Acta.

[15] Li Z.Y., Liu D.P., Liang C.C. (2005). New insight into the molecular mechanisms of MLL-associated leukemia. Leukemia.

[16] Stumpel D.J., Schneider P., van Roon E.H., Boer J.M., de Lorenzo P., Valsecchi M.G., de Menezes R.X., Pieters R., Stam R.W. (2009). Specific promoter methylation identifies different subgroups of MLL-rearranged infant acute lymphoblastic leukemia, influences clinical outcome, and provides therapeutic options. Blood.

[17] Schafer E., Irizarry R., Negi S., McIntyre E., Small D., Figueroa M.E., Melnick A., Brown P. (2010). Promoter hypermethylation in MLL-r infant acute lymphoblastic leukemia: Biology and therapeutic targeting. Blood.

[18] Quinlan C.L., Kaiser S.E., Bolanos B., Nowlin D., Grantner R., Karlicek-Bryant S., Feng J.L., Jenkinson S., Freeman-Cook K., Dann S.G. (2017). Targeting S-adenosylmethionine biosynthesis with a novel allosteric inhibitor of Mat2A. Nat. Chem. Biol..

[19] Ramaswamy S., Tamayo P., Rifkin R., Mukherjee S., Yeang C.H., Angelo M., Ladd C., Reich M., Latulippe E., Mesirov J.P. (2001). Multiclass cancer diagnosis using tumor gene expression signatures. Proc. Natl. Acad. Sci. USA.

[20] Coustan-Smith E., Song G., Clark C., Key L., Liu P., Mehrpooya M., Stow P., Su X., Shurtleff S., Pui C.H. (2011). New markers for minimal residual disease detection in acute lymphoblastic leukemia. Blood.

[21] Armstrong S.A., Staunton J.E., Silverman L.B., Pieters R., den Boer M.L., Minden M.D., Sallan S.E., Lander E.S., Golub T.R., Korsmeyer S.J. (2002). MLL translocations specify a distinct gene expression profile that distinguishes a unique leukemia. Nat. Genet..

[22] Lavallee V.P., Baccelli I., Krosl J., Wilhelm B., Barabe F., Gendron P., Boucher G., Lemieux S., Marinier A., Meloche S. (2015). The transcriptomic landscape and directed chemical interrogation of MLL-rearranged acute myeloid leukemias. Nat. Genet..

[23] Mullighan C.G., Kennedy A., Zhou X., Radtke I., Phillips L.A., Shurtleff S.A., Downing J.R. (2007). Pediatric acute myeloid leukemia with NPM1 mutations is characterized by a gene expression profile with dysregulated HOX gene expression distinct from MLL-rearranged leukemias. Leukemia.

[24] Santos M.A., Faryabi R.B., Ergen A.V., Day A.M., Malhowski A., Canela A., Onozawa M., Lee J.E., Callen E., Gutierrez-Martinez P. (2014). DNA-damage-induced differentiation of leukaemic cells as an anti-cancer barrier. Nature.

[25] Wong P., Iwasaki M., Somervaille T.C., Ficara F., Carico C., Arnold C., Chen C.Z., Cleary M.L. (2010). The miR-17-92 microRNA polycistron regulates MLL leukemia stem cell potential by modulating p21 expression. Cancer Res..

[26] Kaushik S., Liu F., Veazey K.J., Gao G., Das P., Neves L.F., Lin K., Zhong Y., Lu Y., Giuliani V. (2018). Genetic deletion or small-molecule inhibition of the arginine methyltransferase PRMT5 exhibit anti-tumoral activity in mouse models of MLL-rearranged AML. Leukemia.

[27] van Lochem E.G., van der Velden V.H., Wind H.K., te Marvelde J.G., Westerdaal N.A., van Dongen J.J. (2004). Immunophenotypic differentiation patterns of normal hematopoiesis in human bone marrow: Reference patterns for age-related changes and disease-induced shifts. Cytom. B Clin. Cytom..

[28] Boyd K.E., Xiao Y.Y., Fan K., Poholek A., Copeland N.G., Jenkins N.A., Perkins A.S. (2006). Sox4 cooperates with Evi1 in AKXD-23 myeloid tumors via transactivation of proviral LTR. Blood.

[29] Wu S.F., Huang Y., Hou J.K., Yuan T.T., Zhou C.X., Zhang J., Chen G.Q. (2010). The downregulation of onzin expression by PKCepsilon-ERK2 signaling and its potential role in AML cell differentiation. Leukemia.

[30] Zeng C., Xu Y., Xu L., Yu X., Cheng J., Yang L., Chen S., Li Y. (2014). Inhibition of long non-coding RNA NEAT1 impairs myeloid differentiation in acute promyelocytic leukemia cells. BMC Cancer.

[31] Kim J., Piao H.L., Kim B.J., Yao F., Han Z., Wang Y., Xiao Z., Siverly A.N., Lawhon S.E., Ton B.N. (2018). Long noncoding RNA MALAT1 suppresses breast cancer metastasis. Nat. Genet..

[32] Bernt K.M., Zhu N., Sinha A.U., Vempati S., Faber J., Krivtsov A.V., Feng Z., Punt N., Daigle A., Bullinger L. (2011). MLL-rearranged leukemia is dependent on aberrant H3K79 methylation by DOT1L. Cancer Cell.

[33] Ceroi A., Masson D., Roggy A., Roumier C., Chague C., Gauthier T., Philippe L., Lamarthee B., Angelot-Delettre F., Bonnefoy F. (2016). LXR agonist treatment of blastic plasmacytoid dendritic cell neoplasm restores cholesterol efflux and triggers apoptosis. Blood.

[34] Gautier E.L., Westerterp M., Bhagwat N., Cremers S., Shih A., Abdel-Wahab O., Lutjohann D., Randolph G.J., Levine R.L., Tall A.R. (2013). HDL and Glut1 inhibition reverse a hypermetabolic state in mouse models of myeloproliferative disorders. J. Exp. Med..

[35] Bartz F., Kern L., Erz D., Zhu M., Gilbert D., Meinhof T., Wirkner U., Erfle H., Muckenthaler M., Pepperkok R. (2009). Identification of cholesterol-regulating genes by targeted RNAi screening. Cell Metab..

[36] Wheeler K.T., Wang L.M., Wallen C.A., Childers S.R., Cline J.M., Keng P.C., Mach R.H. (2000). Sigma-2 receptors as a biomarker of proliferation in solid tumours. Br. J. Cancer.

[37] Mach R.H., Zeng C., Hawkins W.G. (2013). The sigma2 receptor: A novel protein for the imaging and treatment of cancer. J. Med. Chem..

[38] Moparthi S.B., Arbman G., Wallin A., Kayed H., Kleeff J., Zentgraf H., Sun X.F. (2007). Expression of MAC30 protein is related to survival and biological variables in primary and metastatic colorectal cancers. Int. J. Oncol..

[39] Wang W.T., Han C., Sun Y.M., Chen Z.H., Fang K., Huang W., Sun L.Y., Zeng Z.C., Luo X.Q., Chen Y.Q. (2019). Activation of the Lysosome-Associated Membrane Protein LAMP5 by DOT1L Serves as a Bodyguard for MLL Fusion Oncoproteins to Evade Degradation in Leukemia. Clin. Cancer Res..

[40] Tavor S., Petit I., Porozov S., Avigdor A., Dar A., Leider-Trejo L., Shemtov N., Deutsch V., Naparstek E., Nagler A. (2004). CXCR4 regulates migration and development of human acute myelogenous leukemia stem cells in transplanted NOD/SCID mice. Cancer Res..

[41] Sison E.A., Magoon D., Li L., Annesley C.E., Romagnoli B., Douglas G.J., Tuffin G., Zimmermann J., Brown P. (2015). POL5551, a novel and potent CXCR4 antagonist, enhances sensitivity to chemotherapy in pediatric ALL. Oncotarget.

[42] Daigle S.R., Olhava E.J., Therkelsen C.A., Basavapathruni A., Jin L., Boriack-Sjodin P.A., Allain C.J., Klaus C.R., Raimondi A., Scott M.P. (2013). Potent inhibition of DOT1L as treatment of MLL-fusion leukemia. Blood.

[43] Stumpel D.J., Schneider P., Pieters R., Stam R.W. (2015). The potential of clofarabine in MLL-rearranged infant acute lymphoblastic leukaemia. Eur. J. Cancer.

[44] Lombardini J.B., Sufrin J.R. (1983). Chemotherapeutic potential of methionine analogue inhibitors of tumor-derived methionine adenosyltransferases. Biochem. Pharmacol..

[45] Greco C.M., Powell H.C., Garrett R.S., Lampert P.W. (1980). Cycloleucine encephalopathy. Neuropathol. Appl. Neurobiol..

[46] Lu S.C., Mato J.M. (2012). S-adenosylmethionine in liver health, injury, and cancer. Physiol. Rev..

[47] De La Rosa J., Ostrowski J., Hryniewicz M.M., Kredich N.M., Kotb M., LeGros H.L., Valentine M., Geller A.M. (1995). Chromosomal localization and catalytic properties of the recombinant alpha subunit of human lymphocyte methionine adenosyltransferase. J. Biol. Chem..

[48] Mato J.M., Lu S.C. (2007). Role of S-adenosyl-L-methionine in liver health and injury. Hepatology.

[49] Cao F., Townsend E.C., Karatas H., Xu J., Li L., Lee S., Liu L., Chen Y., Ouillette P., Zhu J. (2014). Targeting MLL1 H3K4 methyltransferase activity in mixed-lineage leukemia. Mol. Cell.

[50] Yvan-Charvet L., Pagler T., Gautier E.L., Avagyan S., Siry R.L., Han S., Welch C.L., Wang N., Randolph G.J., Snoeck H.W. (2010). ATP-binding cassette transporters and HDL suppress hematopoietic stem cell proliferation. Science.

[51] Wang Z., Yip L.Y., Lee J.H.J., Wu Z., Chew H.Y., Chong P.K.W., Teo C.C., Ang H.Y., Peh K.L.E., Yuan J. (2019). Methionine is a metabolic dependency of tumor-initiating cells. Nat. Med..

[52] Serio J., Ropa J., Chen W., Mysliwski M., Saha N., Chen L., Wang J., Miao H., Cierpicki T., Grembecka J. (2018). The PAF complex regulation of Prmt5 facilitates the progression and maintenance of MLL fusion leukemia. Oncogene.

[53] Okada Y., Feng Q., Lin Y., Jiang Q., Li Y., Coffield V.M., Su L., Xu G., Zhang Y. (2005). hDOT1L links histone methylation to leukemogenesis. Cell.

[54] Meyer C., Hofmann J., Burmeister T., Groger D., Park T.S., Emerenciano M., Pombo de Oliveira M., Renneville A., Villarese P., Macintyre E. (2013). The MLL recombinome of acute leukemias in 2013. Leukemia.

[55] Langer T., Metzler M., Reinhardt D., Viehmann S., Borkhardt A., Reichel M., Stanulla M., Schrappe M., Creutzig U., Ritter J. (2003). Analysis of t(9;11) chromosomal breakpoint sequences in childhood acute leukemia: Almost identical MLL breakpoints in therapy-related AML after treatment without etoposides. Genes Chromosomes Cancer.

[56] Reichel M., Gillert E., Angermuller S., Hensel J.P., Heidel F., Lode M., Leis T., Biondi A., Haas O.A., Strehl S. (2001). Biased distribution of chromosomal breakpoints involving the MLL gene in infants versus children and adults with t(4;11) ALL. Oncogene.

[57] Daigle S.R., Olhava E.J., Therkelsen C.A., Majer C.R., Sneeringer C.J., Song J., Johnston L.D., Scott M.P., Smith J.J., Xiao Y. (2011). Selective killing of mixed lineage leukemia cells by a potent small-molecule DOT1L inhibitor. Cancer cell.

[58] Chan-Penebre E., Kuplast K.G., Majer C.R., Boriack-Sjodin P.A., Wigle T.J., Johnston L.D., Rioux N., Munchhof M.J., Jin L., Jacques S.L. (2015). A selective inhibitor of PRMT5 with in vivo and in vitro potency in MCL models. Nat. Chem. Biol..

[59] Duncan K.W., Rioux N., Boriack-Sjodin P.A., Munchhof M.J., Reiter L.A., Majer C.R., Jin L., Johnston L.D., Chan-Penebre E., Kuplast K.G. (2016). Structure and Property Guided Design in the Identification of PRMT5 Tool Compound EPZ015666. ACS Med. Chem. Lett..

[60] Buechele C., Breese E.H., Schneidawind D., Lin C.H., Jeong J., Duque-Afonso J., Wong S.H., Smith K.S., Negrin R.S., Porteus M. (2015). MLL leukemia induction by genome editing of human CD34+ hematopoietic cells. Blood.

[61] Chou T.C. (2006). Theoretical basis, experimental design, and computerized simulation of synergism and antagonism in drug combination studies. Pharmacol. Rev..

[62] Shannon P., Markiel A., Ozier O., Baliga N.S., Wang J.T., Ramage D., Amin N., Schwikowski B., Ideker T. (2003). Cytoscape: A software environment for integrated models of biomolecular interaction networks. Genome Res..

[63] Bushnell B. (2014). BBMap: A Fast, Accurate, Splice-Aware Aligner. https://www.osti.gov/servlets/purl/1241166.

[64] Andrew S. (2010). FastQC: A Quality Control Tool for High Throughput Sequence Data. http://www.bioinformatics.babraham.ac.uk/projects/fastqc.

[65] Dobin A., Davis C.A., Schlesinger F., Drenkow J., Zaleski C., Jha S., Batut P., Chaisson M., Gingeras T.R. (2013). STAR: Ultrafast universal RNA-seq aligner. Bioinformatics.

[66] Love M.I., Huber W., Anders S. (2014). Moderated estimation of fold change and dispersion for RNA-seq data with DESeq2. Genome Biol..

[67] Leek J.T., Johnson W.E., Parker H.S., Jaffe A.E., Storey J.D. (2012). The sva package for removing batch effects and other unwanted variation in high-throughput experiments. Bioinformatics.

