# Supplementary material for: MAT2A as Key Regulator and Therapeutic Target in MLLr Leukemogenesis
Source: Cancers (Basel). 2020 May 24;12(5):1342. doi: 10.3390/cancers12051342 (PMC7281730; doi:10.3390/cancers12051342)
Supplement: Supplementary file 1 [file cancers-12-01342-s001.pdf]

Supplementary Materials

MAT2A as Key Regulator and Therapeutic Target in *MLL*r Leukemogenesis

Kathy-Ann Secker [1], Bianca Bloechl [1], Hildegard Keppeler [1], Silke Duerr-Stoerzer [1], Hannes Schmid [1], Dominik Schneidawind [1], Johan Jeong [2], Thomas Hentrich [3], Julia M. Schulze-Hentrich [3] and Corina Schneidawind [1,*]

[1] Department of Hematology, Oncology, Clinical Immunology and Rheumatology, University Hospital Tuebingen, Tuebingen 72076, Germany; kathy-ann.secker@med.uni-tuebingen.de (K.-A.S.); bianca.bloechl@center1.de (B.B.); hildegard.keppeler@med.uni-tuebingen.de (H.K.); silke.duerr-stoerzer@med.uni-tuebingen.de (S.D.-S.); hannes.schmid@gmx.de (H.S.); dominik.schneidawind@med.uni-tuebingen.de (D.S.)

[2] Synthego Corporation, Menlo Park, CA 94025, USA; jeong401@gmail.com

[3] Institute of Medical Genetics and Applied Genomics, University of Tuebingen, Tuebingen 72076, Germany; thomas.hentrich@med.uni-tuebingen.de (T.H.); julia.schulze-hentrich@med.uni-tuebingen.de (J.M.S.-H.)

* Correspondence: corina.schneidawind@med.uni-tuebingen.de; Tel.: +49-7071-29-84319

Supplementary Methods

Gene expression analyses

Reads were trimmed and cleaned up from contaminating adapters, polyA read through events, and low quality tails with BBDuk of the BBMap [62] tools suite (v38.67) using the following parameters: [k=13 ktrim=r useshortkmers=t mink=5 qtrim=r trimq=10 minlength=20] as well as employing the polyA and truseq_rna k-mer resources. Quality of the cleaned fastq files was assessed using FastQC (v0.11.4) [63, 64] before aligning reads with STAR (v2.7.0a) against the Ensembl H. sapiens genome v95 using the following parameters:[--outFilterType BySJout –outFilterMultimapNmax 20 –alignSJoverhangMin 8 –alignSJDBoverhangMin 1 –outFilterMismatchNmax 999 –outFilterMismatchNoverL max 0.6 –alignIntronMin 20 --outSAMattributes NH HI NM M]. Read counts for all genes were obtained using the featureCounts function of Rsubread (v2.0.0) with [GTF.featureType="exon" GTF.attrType="gene_id" useMetaFeatures=TRUE strandSpecific=1] and DESeq2 (v1.26) [65]. Transcripts covered with <1 read were excluded from subsequent analyses leaving 23.293 genes for determining differential expression. Significance thresholds were set to $| \log2 FC | \geq 0.5$ and BH-adjusted p-value ≤ 0.1. Surrogate variable analysis (sva, v3.26.0) [66] was used to minimize unwanted variation between samples. Differentially expressed genes (DEGs) were examined in downstream analyses for overrepresented canonical pathways and disease aspects with the most recent knowledge base of Ingenuity Pathway Analysis (IPA, v01-16). Only terms with at least three genes were considered. Interactions between DEGs were derived from IPA and visualized with Cytoscape [67].

Supplementary Tables

Table S1. Primer sets for RT-qPCR to quantify relative mRNA expression levels.

Name	Sequence (5´3´)
18S rRNA fwd	CGGCTACCACATCCAAGGAA
18S rRNA rev	GCTGGAATTACCGCGGCT
hu MEIS1 fwd	TGGCCACACGTCACACAGT
hu MEIS1 rev	TTTGTCCTTATCAGGGTCATCATC
hu HOXA9 fwd	ATGAGAGCGGCGGAGACA
hu HOXA9 rev	CGCGCATGAAGCCAGTT
hu MAT2A fwd	TCTTCCAAAGGTTTTGACTACAAGAC
hu MAT2A rev	AGCCAAACATTAAGCCCTGGT
huCDKN1A fwd	TGGAGACTCTCAGGGTCGAAA
huCDKN1A rev	CCGGCGTTTGGAGTGGTA
huLAMP5 fwd	CCAACCTTTTGACATTATCTCAGATTT
huLAMP5 rev	TTGCTCCCGCTCATCCA
huCXCR4 fwd	TGGAGGGGATCAGTATATACACTTCA
huCXCR4 rev	TCATAGTCCCCTGAGCCCATT
huCDK1 fwd	CCATTGACTAACTATGGAAGATTATACCA
huCDK1 rev	TGTCTACCCTTATACACAACTCCATAGG

Table 2. Most differentially expressed genes of *MLL-AF4* cells treated with PF-9366 compared to DMSO control. *MLL-AF4* cells were treated with 15 μM of PF-9366 or vehicle (DMSO) for 4 days and two biological replicates (n = 2) were used for RNA-seq. The top up- and down-regulated genes ranked by adjusted p-value are shown.

Gene	logFC	Adj. p-value
VCAN	-1.75424241	2.3502E-17
MALAT1	1.23960918	5.5077E-15
ABCG1	4.53786533	6.8832E-12
NEAT1	1.54858668	3.6131E-11
CDKN1A	1.63818129	2.5831E-10
TARBP1	1.59297755	2.7678E-09
IFI6	1.54993958	8.3779E-08
PLAC8	-1.31980931	8.3779E-08
MT2A	-2.17730764	1.1129E-07
SOX4	-1.15971622	1.1281E-06
CXCR4	-0.94062791	2.1118E-06
ACPP	-1.89200163	3.9511E-06
PLIN2	1.19252527	7.5285E-06
CTSC	-1.03995125	9.7484E-06
MT-TL1	0.96366607	4.3491E-05
MAT2A	1.04809981	6.6571E-05
MT1H	-3.58994646	9.1747E-05
TYMP	0.91988146	0.00017035
MT-ND6	0.93029829	0.00017035
GABPB1-AS1	1.16341383	0.00023592
ACAT2	-1.02169596	0.00039853
ANP32E	-0.92615736	0.00105122
OAZ1	-0.67269662	0.00105122
ABCA1	3.0159732	0.00163224
IRF8	-0.91266434	0.00181206
HMGB2	-0.73968999	0.00215973
C19orf38	-2.13047762	0.00335439
TMEM97	-1.25392963	0.00394383
FGD2	-2.05658855	0.00578347
MAN2A2	1.07818151	0.00618254
LINC00685	2.01729224	0.00813506
CD14	0.70081371	0.00881832
NGFR	-3.0884782	0.0089542
MYLIP	0.87693769	0.00903504
VIM	-0.70787279	0.01359915
RB1	-1.27508179	0.01359915

PLTP	1.04604357	0.01359915
LAPTM5	0.67118504	0.01690308
MT1X	-1.56656477	0.019355
SRRM2	0.58245028	0.01935625
FASN	0.66011712	0.01935625
BCAT1	-0.86734093	0.0226492
SAPCD2	-0.82305676	0.02379786
FTH1	0.61891478	0.02473858
ACTA2	1.74733019	0.02473858
ETV1	-3.27142883	0.02533037
PSAT1	-0.95821737	0.02533037
JUN	0.94619992	0.02547328
MT1G	-2.49105465	0.03788124
IRS2	0.70454355	0.03864554
HTRA3	1.13739561	0.04771929
TGFBI	-0.76339965	0.05345073
CTSB	0.60951319	0.05450355
GOLGA8A	1.13291029	0.05450355
GGT5	-2.08399121	0.05450355
SLC20A1	0.71301397	0.05616531
STMN1	-0.55588437	0.06043213
TMEM158	2.21094617	0.06052583
CLEC5A	1.85066641	0.06739138
MDK	-3.43802962	0.06739138
MDM2	0.59029142	0.06739138
WSB1	0.6121163	0.06739138
LAMP5	-0.61469936	0.06739138
CPNE7	1.65893052	0.07569192
RNU4ATAC	-1.5679689	0.07666438
RPS27L	0.51840689	0.08337264
TFRC	0.65673972	0.0943774
CDK1	-0.72323939	0.0943774
FURIN	0.69614509	0.0943774
KCTD5	-0.82984697	0.0943774
FDPS	-0.59437789	0.09754882
DPYSL3	-1.21154341	0.09754882
WASF1	0.8929807	0.09786513
ALDOC	-1.11734961	0.09805786

Supplementary Figures

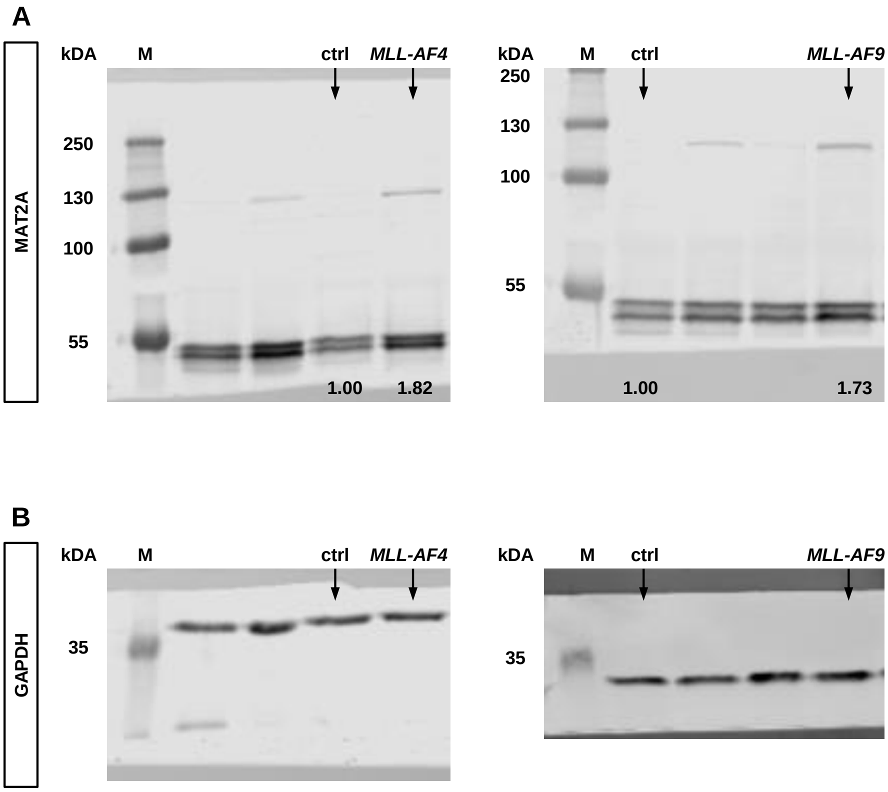

Figure S1. Expanded data for western blots including molecular weight marker as already shown in Figure 1C. Uncropped data figure for images shown in Figure 1C. Representative western blot analysis shows increased MAT2A expression in *MLL*r cells compared to culture expanded CD34+ huCB control cells. MAT2A protein is shown in **(A)**. Glyceraldehyde-3-phosphate dehydrogenase (GAPDH) was used as loading control **(B)**.

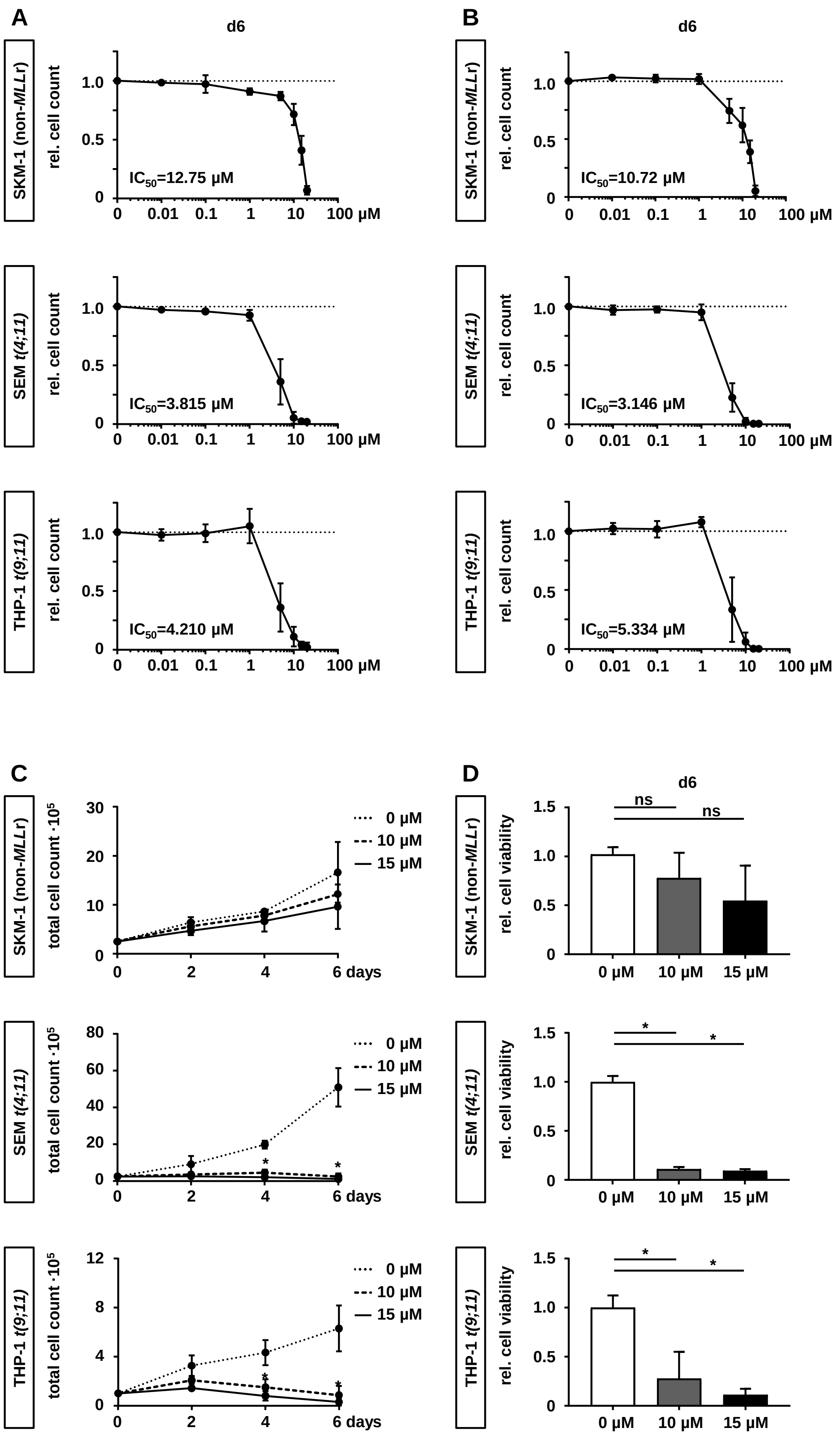
A
B
C
D
d6
SKM-1 (non-MLLr)
SEM t(4;11)
THP-1 t(9;11)
rel. cell count
IC50=12.75 µM
IC50=3.815 µM
IC50=4.210 µM
IC50=10.72 µM
IC50=3.146 µM
IC50=5.334 µM
0 0.01 0.1 1 10 100 µM
total cell count ·10^5
0 2 4 6 days
0 µM
10 µM
15 µM
rel. cell viability
ns
*

Figure S2. PF-9366 preferentially suppresses proliferation and viability of *MLL*r cell lines. (A) *MLL*r cell lines SEM and THP-1 or non-*MLL*r cell line SKM-1 were treated with increasing concentrations of PF-9366 or vehicle (DMSO) for 6 days and relative cell count was determined by staining with Trypan blue. Pooled data of three biological replicates (n = 3) performed in technical triplicates are shown. Calculated IC_{50} values are indicated. **(B)** Relative cell count was further confirmed by flow cytometry using counting beads. Pooled data of three biological replicates (n = 3) performed in technical triplicates are shown. Calculated IC_{50} values are indicated. **(C)** To assess proliferation curves, cell lines were again treated with PF-9366 (10 μM, 15 μM) or vehicle (DMSO) and total cell count was determined by Trypan blue staining every second day. The mean of pooled data of three biological replicates (n = 3) performed in technical triplicates is shown. One-way ANOVA was used with Sidak correction: *, $p < 0.05$. **(D)** Cell viability of cell lines upon inhibition treatment with PF-9366 (10 μM, 15 μM) or control (DMSO) was determined after 6 days via fluorescence-based read of alamarBlue assay. Experiment was performed in three biological replicates (n = 3) and technical triplicates. Bars represent the mean. Error bars indicate SD. One-way ANOVA was used with Sidak correction: *, $p < 0.05$.

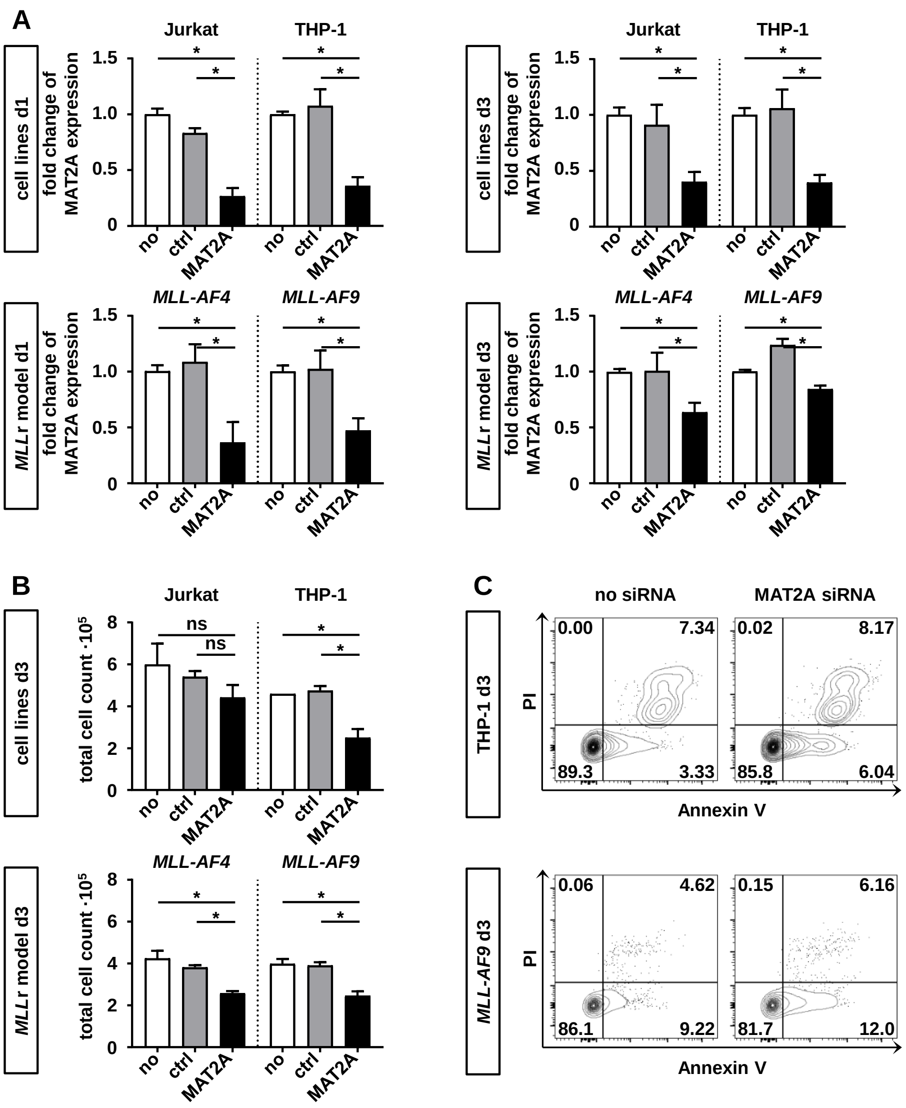

Figure S3. siRNA-mediated *MAT2A* knock-down.(A) Jurkat cells (non-*MLL*r), THP-1 (*MLL-AF9*) and CRISPR/Cas9-*MLL*r cells with *t*(4;11) and *t*(9;11) were nucleofected with MAT2A siRNA, non-target control siRNA (ctrl) or no siRNA (no). Fold change of *MAT2A* expression measured by RT-qPCR is displayed for day 1 and day 3 after nucleofection. Pooled data of two independent experiments (n = 2) performed in technical triplicates are shown. Bars represent the mean. Error bars indicate SD. One-way ANOVA was used with Sidak correction: *, $p < 0.05$. **(B)** Total cell amount was microscopically determined 3 days after nucleofection by Trypan blue staining. Pooled data of two independent

experiments (n = 2) performed in technical duplicates are shown. Bars represent the mean. Error bars indicate SD. One-way ANOVA was used with Sidak correction: *, $p < 0.05$. **(C)** Representative flow cytometry analyses of apoptotic cells 3 days after *MAT2A* siRNA knock-down determined by Annexin V staining are shown.

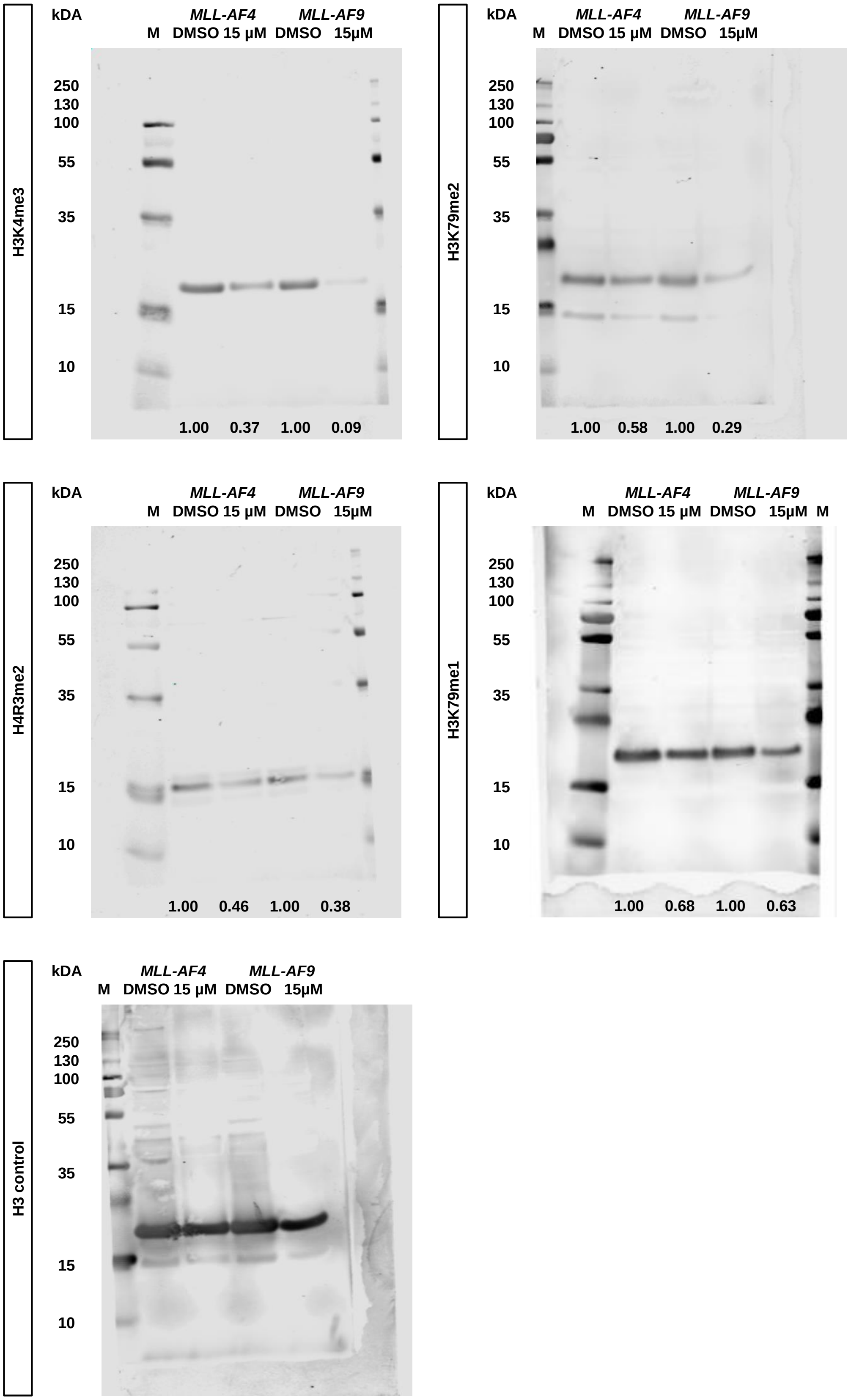
H3K4me3
kDA
MLL-AF4 MLL-AF9
M DMSO 15 μM DMSO 15μM
250
130
100
55
35
15
10
1.00 0.37 1.00 0.09
H3K79me2
kDA
MLL-AF4 MLL-AF9
M DMSO 15 μM DMSO 15μM
250
130
100
55
35
15
10
1.00 0.58 1.00 0.29
H4R3me2
kDA
MLL-AF4 MLL-AF9
M DMSO 15 μM DMSO 15μM
250
130
100
55
35
15
10
1.00 0.46 1.00 0.38
H3K79me1
kDA
MLL-AF4 MLL-AF9
M DMSO 15 μM DMSO 15μM M
250
130
100
55
35
15
10
1.00 0.68 1.00 0.63
H3 control
kDA
MLL-AF4 MLL-AF9
M DMSO 15 μM DMSO 15μM
250
130
100
55
35
15
10

Figure S4. Expanded data for western blots including molecular weight marker as already shown in Figure 2D. Uncropped data figure for images shown in Figure 2D. Representative western blot analysis shows decreased histone methylation in *MLL*r cells upon PF-9366 treatment. Histone H3 was used as loading control.

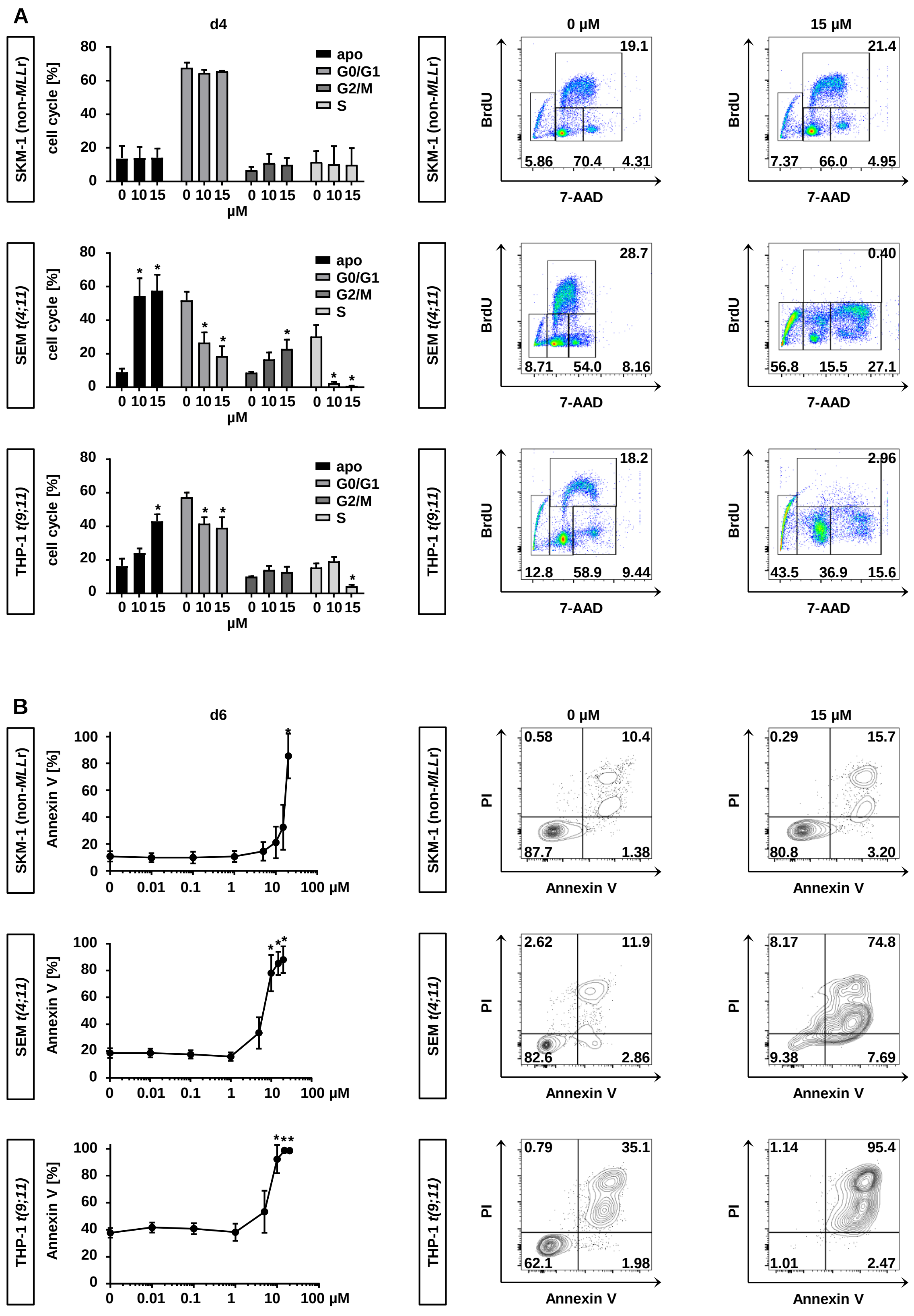
A
d4
SKM-1 (non-MLLr)
SEM t(4;11)
THP-1 t(9;11)
cell cycle [%]
apo
G0/G1
G2/M
S
0 10 15
µM
0 µM
15 µM
BrdU
7-AAD
19.1
5.86
70.4
4.31
21.4
7.37
66.0
4.95
28.7
8.71
54.0
8.16
0.40
56.8
15.5
27.1
18.2
12.8
58.9
9.44
2.96
43.5
36.9
15.6
B
d6
Annexin V [%]
0
0.01
0.1
1
10
100 µM
PI
Annexin V
0.58
10.4
87.7
1.38
0.29
15.7
80.8
3.20
2.62
11.9
82.6
2.86
8.17
74.8
9.38
7.69
0.79
35.1
62.1
1.98
1.14
95.4
1.01
2.47

Figure S5. Inhibition of MAT2A diminishes DNA synthesis and enhances apoptosis of *MLL*r cell lines. *MLL*r cell lines SEM and THP-1 or non-*MLL*r cell line SKM-1 were treated with PF-9366 (10 µM, 15 µM) or vehicle (DMSO). **(A)** Pooled data (left) from three biological replicates (n = 3) performed in technical triplicates and representative (right) cell cycle analysis of single cells after 4 days is shown. Data were acquired using BrdU staining and flow cytometry. **(B)** Annexin V staining revealed a dose-dependent translocation of phosphatidylserine to the outer leaflet of the plasma membrane in apoptotic cells after 6 days of treatment. Experiment was performed in three biological replicates (n = 3) and technical triplicates. Pooled (left) and representative (right) data are shown. Bars or dots represent the mean. Error bars indicate SD. One-way ANOVA was used with Dunnett correction: *, $p < 0.05$.

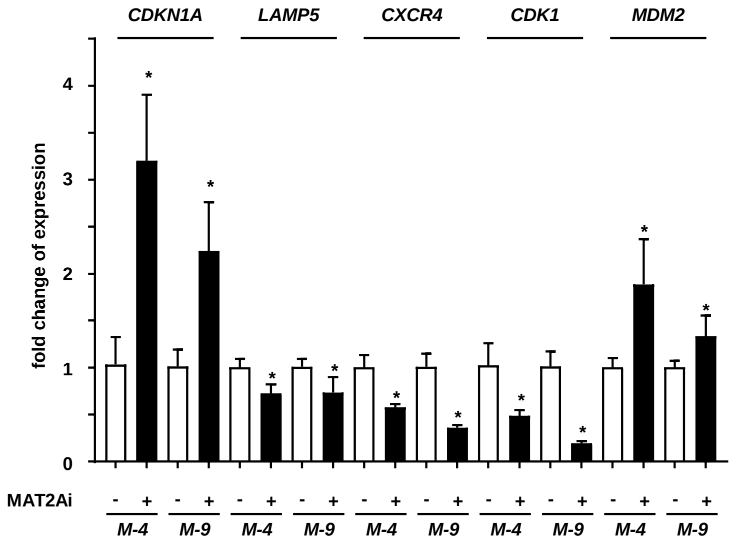

Figure S6: Validation of RNA-seq data via RT-qPCR. RNA-seq data displayed in Figure 5 were validated using RT-qPCR in CRISPR/Cas9-*MLL-AF4* (*M-4*) and -*AF9* (*M-9*) cells. Bars represent the mean. Error bars indicate SD. *Student's* t-test was used: *, $p < 0.05$.

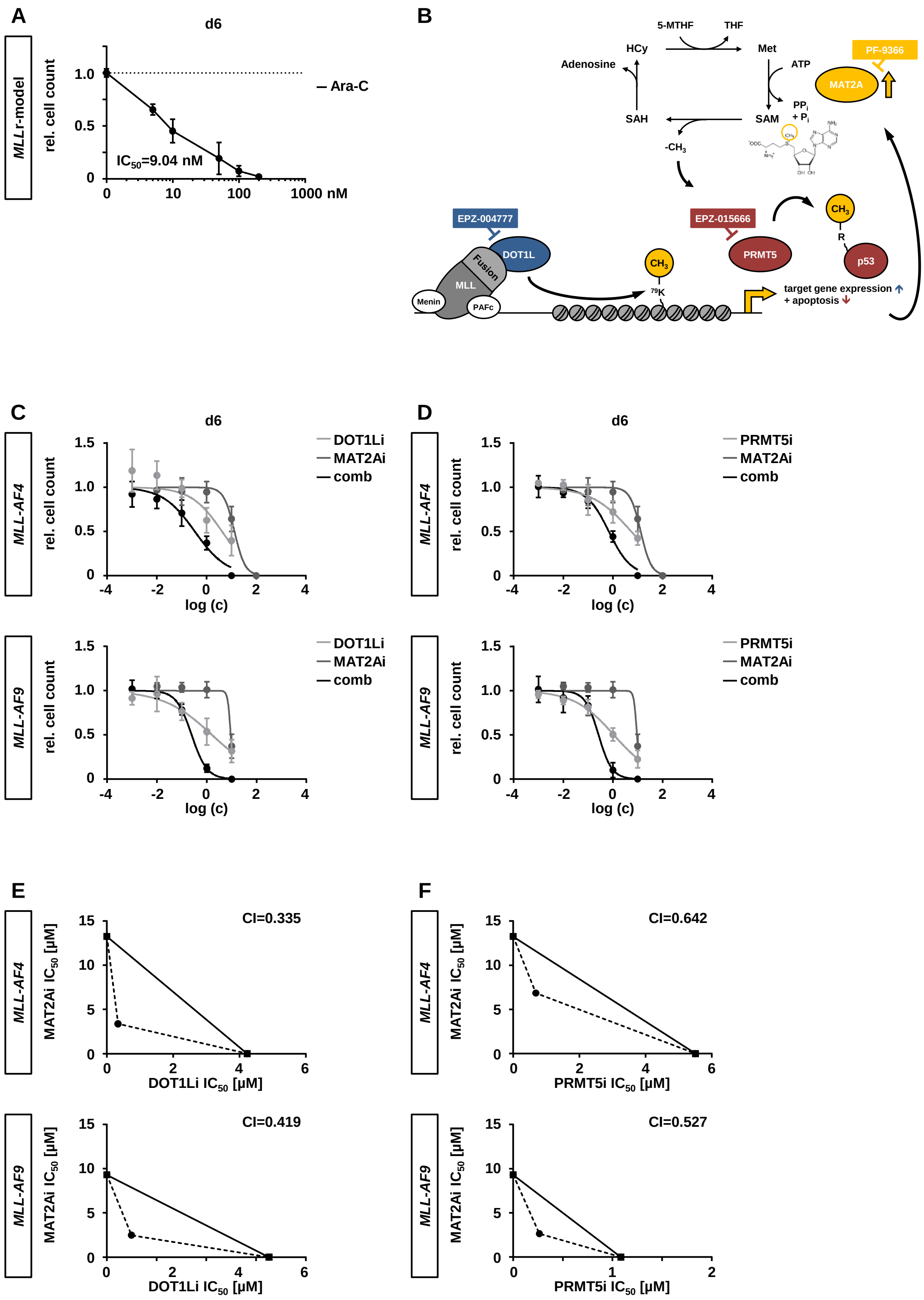
A
d6
MLLr-model
rel. cell count
1.0
0.5
0
IC50=9.04 nM
0
10
100
1000 nM
Ara-C
B
5-MTHF
THF
HCy
Met
Adenosine
ATP
SAH
SAM
PPi + Pi
-CH3
PF-9366
MAT2A
EPZ-004777
DOT1L
EPZ-015666
PRMT5
CH3
R
p53
Fusion
MLL
Menin
PAFc
79K
target gene expression
+ apoptosis
C
d6
MLL-AF4
MLL-AF9
rel. cell count
1.5
1.0
0.5
0
-4
-2
0
2
4
log (c)
DOT1Li
MAT2Ai
comb
D
d6
PRMT5i
MAT2Ai
comb
E
CI=0.335
CI=0.419
MAT2Ai IC50 [µM]
15
10
5
0
2
4
6
DOT1Li IC50 [µM]
F
CI=0.642
CI=0.527
PRMT5i IC50 [µM]
1
2

Figure S7. Combinational treatment with PF-9366 and chemotherapy or targeted therapy augments anti-leukemic effects. (A) *MLL-AF4*/*-AF9* cells were treated with increasing concentrations of cytarabine (Ara-C) for 6 days and cell count was determined by flow cytometry using counting beads. The mean of two biological replicates (n = 2) performed in technical triplicates is shown. Calculated IC50 value is indicated. (B) A scheme of the *MLL*r dependent MAT2A pathomechanism with SAM within the metabolic pathway influencing DOT1L and PRMT5 resulting in aberrant histone, DNA or protein methylation is displayed. (C) *MLL-AF4*/*-AF9* cells were treated with increasing concentrations of EPZ004777 (DOT1Li) or (D) EPZ015666 (PRMT5i) and PF-9366 (MAT2Ai) for dose-response curves to determine IC50 values of single treatments. Additionally, dose-response curves of the combinational treatment with MAT2Ai at a constant drug ratio of 1:10 were analyzed. Relative cell count was determined by flow cytometry using counting beads and the mean is displayed. Experiments were performed in three biological replicates (n = 3) and technical triplicates. IC50 values are indicated. Error bars indicate SD. For synergy calculation, IC50 values were used to display isobolograms at 50% inhibition levels and to calculate combination indices (CI) displayed in (E) and (F). CI < 1 indicates synergy.

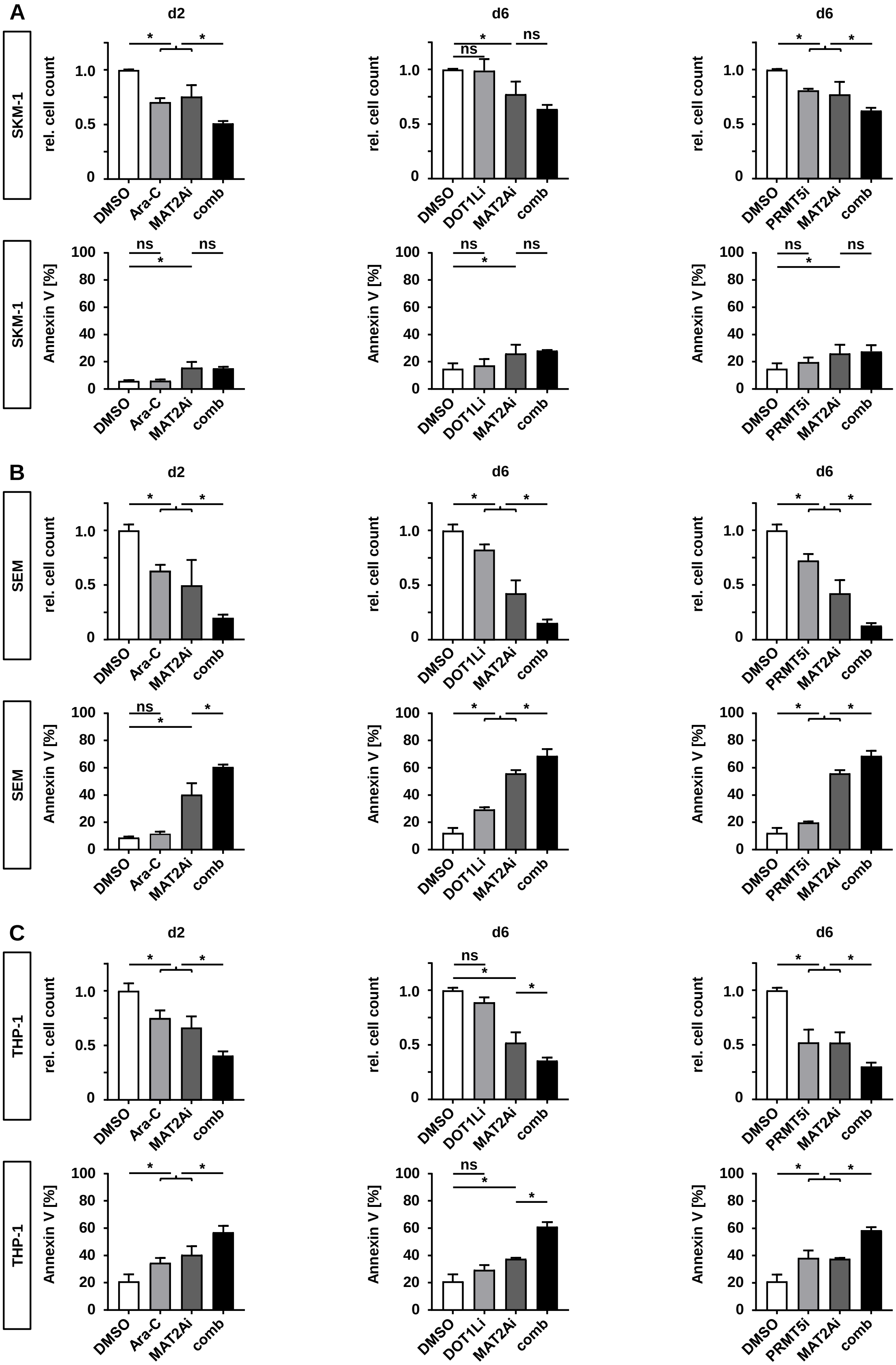
A
B
C
SKM-1
SEM
THP-1
d2
d6
rel. cell count
Annexin V [%]
DMSO
Ara-C
DOT1Li
PRMT5i
MAT2Ai
comb
ns
*
0
0.5
1.0
20
40
60
80
100

Figure S8. PF-9366 in combination with chemotherapy or targeted therapies leads to an *MLL*r-specific additive effect validated with cell lines. Cell lines SKM-1 **(A)**, SEM **(B)**, THP-1 **(C)** were treated with 10 nM cytarabine (Ara-C), 10 μM PF-9366 (MAT2Ai), 1 μM DOT1Li (EPZ004777) and 1 μM PRMT5i (EPZ015666) either as single agents or simultaneously for 2 or 6 days as indicated. Vehicle (DMSO) was used as control. Relative cell count was determined by flow cytometry using counting beads. Experiment was performed in three biological replicates (n = 3) and technical duplicates. Apoptotic cells were detected using Annexin V staining after 6 days of respective treatments. Pooled data of three biological replicates (n = 3) performed in technical duplicates are shown. Data were acquired using flow cytometry. Bars represent the mean. Error bars indicate SD. One-way ANOVA was used with Sidak correction: *, $p < 0.05$.

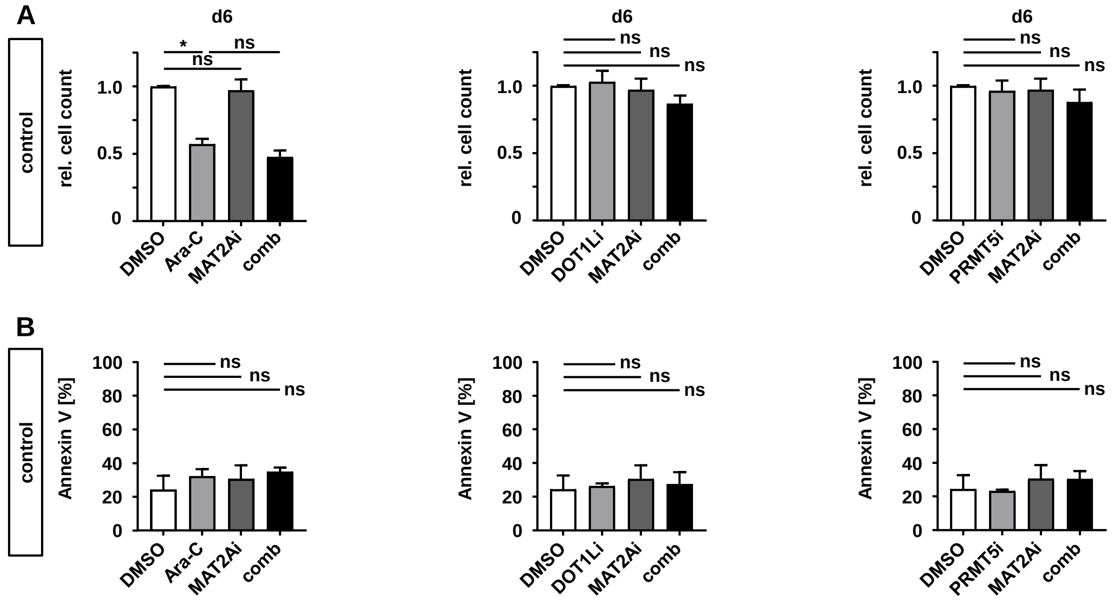

Figure S9. PF-9366 alone or in combination with targeted therapies has no negative impact on control cells. CD34+ control cells were treated with 10 nM cytarabine (Ara-C), 10 µM PF-9366 (MAT2Ai), 1 µM DOT1Li (EPZ004777) and 1 µM PRMT5i (EPZ015666) either as single agents or simultaneously for 6 days. Vehicle (DMSO) was used as control. **(A)** Relative cell count was determined by flow cytometry using counting beads. Experiment was performed in three biological replicates (n = 3) and technical duplicates. **(B)** Apoptotic cells were detected using Annexin V staining after 6 days of respective treatments. Pooled data of three biological replicates (n = 3) performed in technical duplicates are shown. Data were acquired using flow cytometry. Bars represent the mean. Error bars indicate SD. One-way ANOVA was used with Dunnett correction: *, $p < 0.05$.
